# Morphology and osteo‐histology of the weigeltisaurid wing: Implications for aerial locomotion in the world's first gliding reptiles

**DOI:** 10.1111/joa.70058

**Published:** 2025-10-27

**Authors:** Valentin Buffa, Jordan Gônet, Thomas van de Kamp, Marcus Zuber, Marc Girondot, Eberhard Frey, J.‐Sébastien Steyer, Michel Laurin

**Affiliations:** ^1^ Evolutionary Studies Institute University of the Witwatersrand Johannesburg South Africa; ^2^ Centre de Recherche en Paléontologie – Paris, UMR 7207 CNRS‐MNHN‐SU, Muséum National d'Histoire Naturelle Paris France; ^3^ UMR 7179: Mécanismes Adaptatifs & évolution (MECADEV), CNRS‐MNHN Paris France; ^4^ Karlsruhe Institute of Technology (KIT), Institute for Photon Science and Synchrotron Radiation (IPS) Eggenstein‐Leopoldshafen Germany; ^5^ Laboratory for Applications of Synchrotron Radiation (LAS) Karlsruhe Institute of Technology (KIT) Karlsruhe Germany; ^6^ Laboratoire Écologie, Société, Évolution, Université Paris‐Sud, AgroParisTech, CNRS, Université Paris Saclay Gif‐sur‐Yvette France; ^7^ Karlsruhe Institute of Technology (KIT) Institute for Geography and Geology (IfGG) Karlsruhe Germany

**Keywords:** gastralia, gliding, histology, patagium, Permian, Weigeltisauridae

## Abstract

The Late Permian Weigeltisauridae are the world's first gliding reptiles, but much remains unknown regarding the anatomy of their patagium (or wing), which, in turn, confounds our understanding of their gliding mechanism and paleobiology. Here, we examine the morphology and osteo‐histology of the patagial skeleton of weigeltisaurids using an array of imaging techniques and several thin sections through the wing skeleton of a specimen of *Weigeltisaurus* from the Late Permian of Germany. We demonstrate that patagials and gastralia share a one‐to‐one articulation, permitted by the uniquely specialized anatomy of the lateral gastralia. We also show, based on skeletal anatomy, histology, and inferred musculoskeletal relationships, that patagials are likely neomorphic ossifications and are thus not strictly homologous to the gastralia. We provide the first reconstruction of the musculoskeletal anatomy of the weigeltisaurid wing, suggesting that the base of the patagials was likely embedded in the M. obliquus externus group. Similar to the condition in the extant flying lizard *Draco*, these muscles may have contributed to the unfolding of the patagium, which was likely supported by hooking the manual claws onto the leading edge of the patagium. This would have provided weigeltisaurids with a means to maintain the patagium expanded and under tension while gliding, as well as some measure of control of the dihedral angle of the wing, thereby offering a means to control stability and maneuverability in flight. Wing folding may have been permitted by muscular and tendinous connections between the elements of the patagial skeleton, generating elastic tension toward a folded state, as in *Draco*. Lastly, the cross sections of the patagials show a bimodal cortical distribution with much thicker cortices along their cross‐sectional long axis than short axis. This made the patagials rigid, which likely helped prevent patagial collapse during gliding. This work represents a critical step toward understanding the wing structure and gliding mechanism in weigeltisaurids, paving the way for future morphofunctional or biomechanical studies on the locomotion of the world's first flying vertebrates.

## Graphical abstract

This study investigates the morphology and osteo‐histology of the wing skeleton of the world's first gliding reptiles, showing how it differs from those of extant gliding lizards, yet is also convergently similar. These findings pave the way for future biomechanical studies on the gliding locomotion of these emblematic fossil animals.

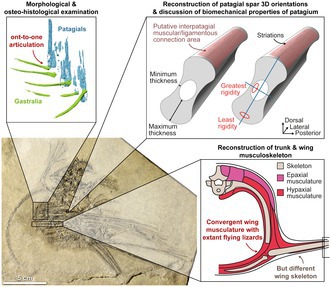

## 1 INTRODUCTION

The evolution of new locomotor adaptations has long been considered to be linked to increased morphological evolution and ecological and specific diversity (Schluter, 2000). These innovations often rely on anatomical modifications of the skeleton in vertebrates that can in turn be identified in extinct taxa (e.g., Fröbisch & Reisz, 2009; Kelley & Pyenson, 2015; Padian, 1985). In gliding vertebrates, this often takes the form of morphological specializations enabling them to use their body or parts of it as an airfoil to perform a controlled descent and move around safely in the complex forest canopy environments they live in (Dudley et al., 2007; Khandelwal et al., 2023; Socha et al., 2015). For instance, the gliding squamates of the genus *Draco* of South‐East Asia bear broad membranous patagia supported by extremely elongate and flexible trunk ribs (Colbert, 1967; McGuire & Dudley, 2011; Russell & Dijkstra, 2001) that serve as lift generating surfaces (Buffa, Salaün, & Cinnella, 2024b; Khandelwal & Hedrick, 2022; Lau et al., 2023). A patagium is a partly membranous structure that generates aerodynamic lift when exposed to an airflow and allows flight. Patagia often stretch between the body and/or limbs in various animals, although they can also be supported by other skeletal elements, such as the dorsal ribs in *Draco*. Incidentally, several unrelated taxa of extinct reptiles show strikingly similar rib‐supported patagia, such as *Xianglong* from the Early Cretaceous of China (Li et al., 2007) and the enigmatic *Mecistotrachelos* (Fraser et al., 2007) and kuehneosaurids (Colbert, 1970; Stein et al., 2008) from the late Triassic of Europe and North America.

The first occurrence of putative gliding reptiles dates back to the Weigeltisauridae, known from the Late Permian of Germany, England, Russia, and Madagascar (Buffa et al., 2021, 2022; Bulanov & Sennikov, 2010; Pritchard et al., 2021). These reptiles are considered as non‐saurian neodiapsids, that is, close relatives of the reptile crown group (Buffa et al., 2025; Buffa & Laurin, 2024; Pritchard et al., 2021). This group comprises five nominal species in four genera: *Coelurosauravus elivensis* Piveteau, 1926; *Glaurung scheideri* Bulanov & Sennikov, 2015; *Rautiania alexandri* Bulanov & Sennikov, 2006; *Rautiania minichi* Bulanov & Sennikov, 2006; and *Weigeltisaurus jaekeli* (Weigelt, 1930). *Wapitisaurus problematicus* Brinkman, 1988, from the Early Triassic of Canada was also considered as a weigeltisaurid but was recently reinterpreted as a thalattosauroid thallatosaur (Bastiaans et al., 2023).

In contrast to other reptilian gliders with rib‐supported patagia such as *Draco*, the patagia of weigeltisaurids are supported by a series of elongate bony rods called patagial spars or patagials that emerge from the lateral wall of the trunk (Buffa et al., 2022; Frey et al., 1997; Pritchard et al., 2021; Schaumberg et al., 2007). Although outwardly similar, the patagial skeleton of weigeltisaurids is thus markedly different from that of all other known gliding reptiles and is also unique even compared to all other vertebrate gliders. However, the nature of the weigeltisaurid patagial skeleton remains elusive and debated.

Upon its discovery, the holotype of *Weigeltisaurus jaekeli* (Weigelt, 1930) from the Late Permian of Germany was initially informally identified as a “flying reptile” (“Flugsaurier”; Weigelt, 1930, p. 41) because of its patagial skeleton. However, these bones were subsequently reinterpreted as coelacanth fin rays and mostly removed during preparation to expose the skeleton of the trunk and limbs. The flying capacity of this animal was thus dismissed (Weigelt, 1930). At about the same time, *Coelurosauravus elivensis* Piveteau, 1926 was described from the Late Permian of Madagascar, but none of the specimens referred by Piveteau (1926) preserve a substantial portion of the patagial skeleton. The gliding ability of *C. elivensis* was thus also overlooked at the time.

The putative gliding capabilities of weigeltisaurids were only recognized decades later (Carroll, 1978; Evans, 1982; Evans & Haubold, 1987; Pettigrew, 1979; Schaumberg, 1976, 1986), but raised the question of the homology of the patagial skeleton of weigeltisaurids. Carroll (1978) identified these bones as elongate trunk ribs, and Pettigrew (1979), Evans (1982), and Evans and Haubold (1987) identified them as the distal portion of bipartite trunk ribs, whereas Schaumberg (1976, 1986) considered the patagials as part of the dermal skeleton. Subsequent studies supported the latter interpretation and suggested that the patagials are neomorphic dermal ossifications (Frey et al., 1997; Schaumberg et al., 2007). More recently, Pritchard et al. (2021) identified three possible interpretations for the homology of the patagials: (i) intermuscular ossifications that formed within the myosepta of external trunk musculature; (ii) modified lateral gastralia; (iii) true neomorphs without homology with any pre‐existing soft or hard tissues. These authors tentatively interpreted the patagials as true neomorphs, whereas Buffa et al. (2022) tentatively identified them as modified lateral gastralia. Both studies concur on the close topographical relationship between the patagials and the gastral basket, indicating that the patagium originated from the ventrolateral margin of the trunk, ventral to the center of gravity of the animal (Buffa et al., 2022; Pritchard et al., 2021). Yet, the articulation between the patagials and the rest of the skeleton remains unclear.

In this context, the osteological nature of the patagials remains debated, and much remains unclear of the overall organization of the patagial skeleton despite several re‐examinations of weigeltisaurid anatomy (Buffa et al., 2022; Pritchard et al., 2021; Schaumberg et al., 2007). Similarly, very little is known regarding the musculoskeletal relationships of the patagials (discussed in Pritchard et al., 2021). Lastly, little to no examination has been conducted on the biomechanical properties of the patagials and their bearing on gliding performance. It is thus necessary to turn to other methods to study the enigmatic patagial skeleton of weigeltisaurids. Paleohistology is a reliable method to assess the nature and function of unusual skeletal elements (e.g., Cerda et al., 2022; Klein et al., 2012; Rytel et al., 2024) and could provide new insight into these bones and weigeltisaurid paleobiology in general.

Schaumberg et al. (2007) produced the only histological thin sections of weigeltisaurid material to date: a transverse section of a patagial, a longitudinal section of a patagial, and a transverse section of a femur, all from a single specimen from the Brandt private collection. However, these sections were only briefly discussed (Canoville et al., 2021; Schaumberg et al., 2007) and do not illustrate the histology and microstructure of the full extent of the wing skeleton.

Here, we examine the external morphology, microstructure, and histology of the weigeltisaurid patagial skeleton based on detailed observations of most specimens available in public collections, tomographic and laminographic X‐ray scan data, as well as a series of thin sections through a partial wing of *Weigeltisaurus* sp. from the Late Permian of Germany. In light of this new anatomical data, this study thus aims to provide the most detailed investigation of the weigeltisaurid wing to date in order to reconstruct its patagial and trunk skeleton and musculature, and to understand the homology of the patagials. This will in turn allow for the first assessment of the biomechanical properties of the weigeltisaurid wing in the context of gliding locomotion.

## 2 MATERIALS AND METHODS

### 2.1 Materials examined

This study is mostly based on the examination of the patagial skeleton of two specimens from the Late Permian of Germany, SMNK‐PAL 2882 and SMNK‐PAL 34865. Both specimens come from the Kupferschiefer Formation, the lowest unit of the Zechstein Group. This formation is generally considered to be of Lopingian age, although the exact age (Wuchiapingian or Changhsingian) is debated (summarized in Pritchard et al., 2021).

**FIGURE 1:**
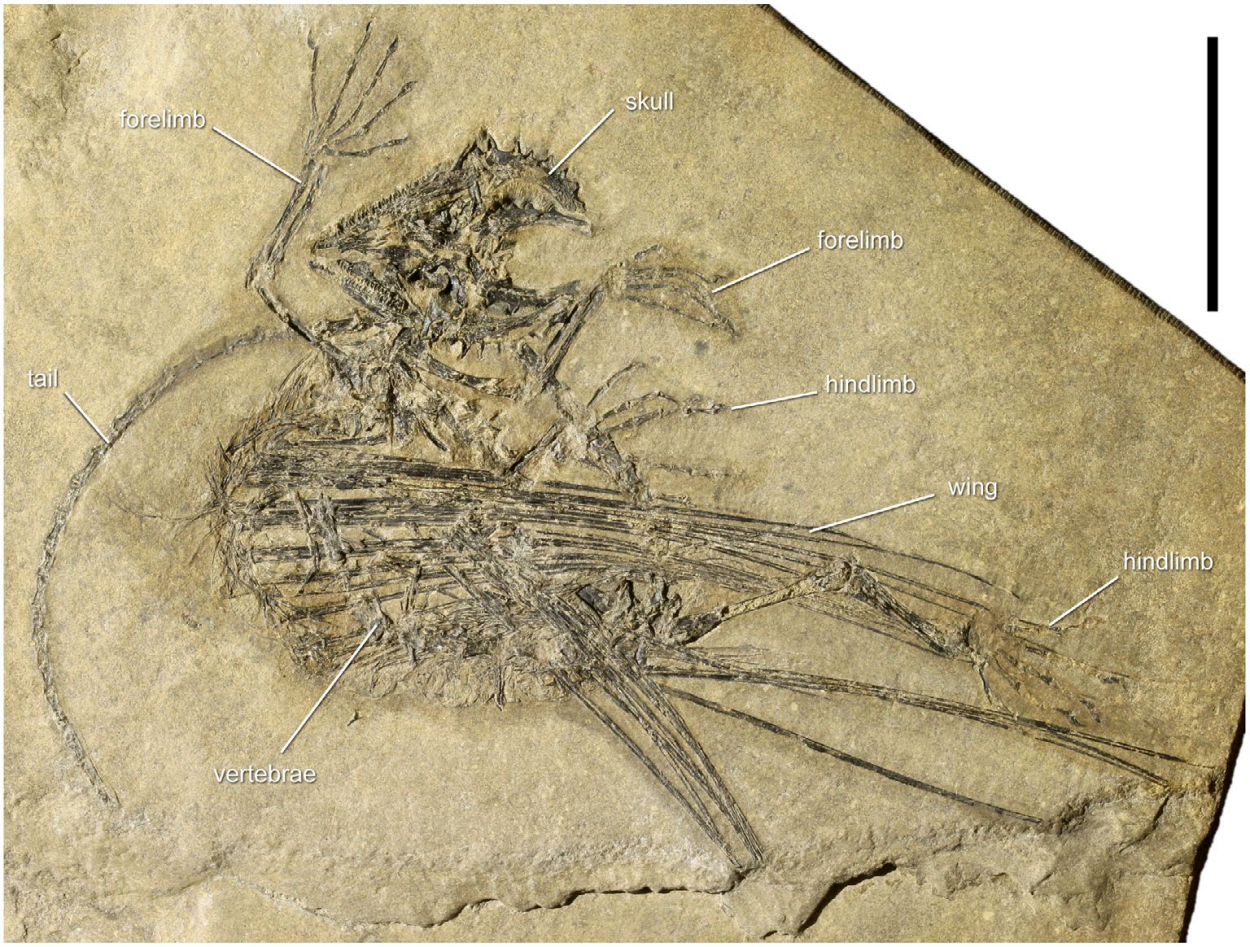
*Weigeltisaurus* sp. Kuhn, 1939 (Germany, Wuchiapingian), Ellrich specimen SMNK‐PAL 2882. Scale bar, 50 mm.

SMNK‐PAL 2882 is an exquisitely preserved, nearly complete articulated specimen from the Ellrich locality in the Mansfeld district (Saxony‐Anhalt, Germany) (Figure 1). The bones of the specimen are strongly compressed dark brown to black and preserved on part and counterpart slabs of a finely laminated shale of light brownish‐grey color. The main part is housed in the SMNK, whereas the counterpart is held in a private collection and unavailable for scientific study. This specimen was initially described by Frey et al. (1997), subsequently by Schaumberg et al. (2007), and more recently by Pritchard et al. (2021). The latter authors referred this specimen to the species *Weigeltisaurus jaekeli*, but we follow here Buffa et al.'s (2021, 2022) delimitation of this species and provisionally refer the so‐called Ellrich specimen to *Weigeltisaurus* sp. instead.

**FIGURE 2:**
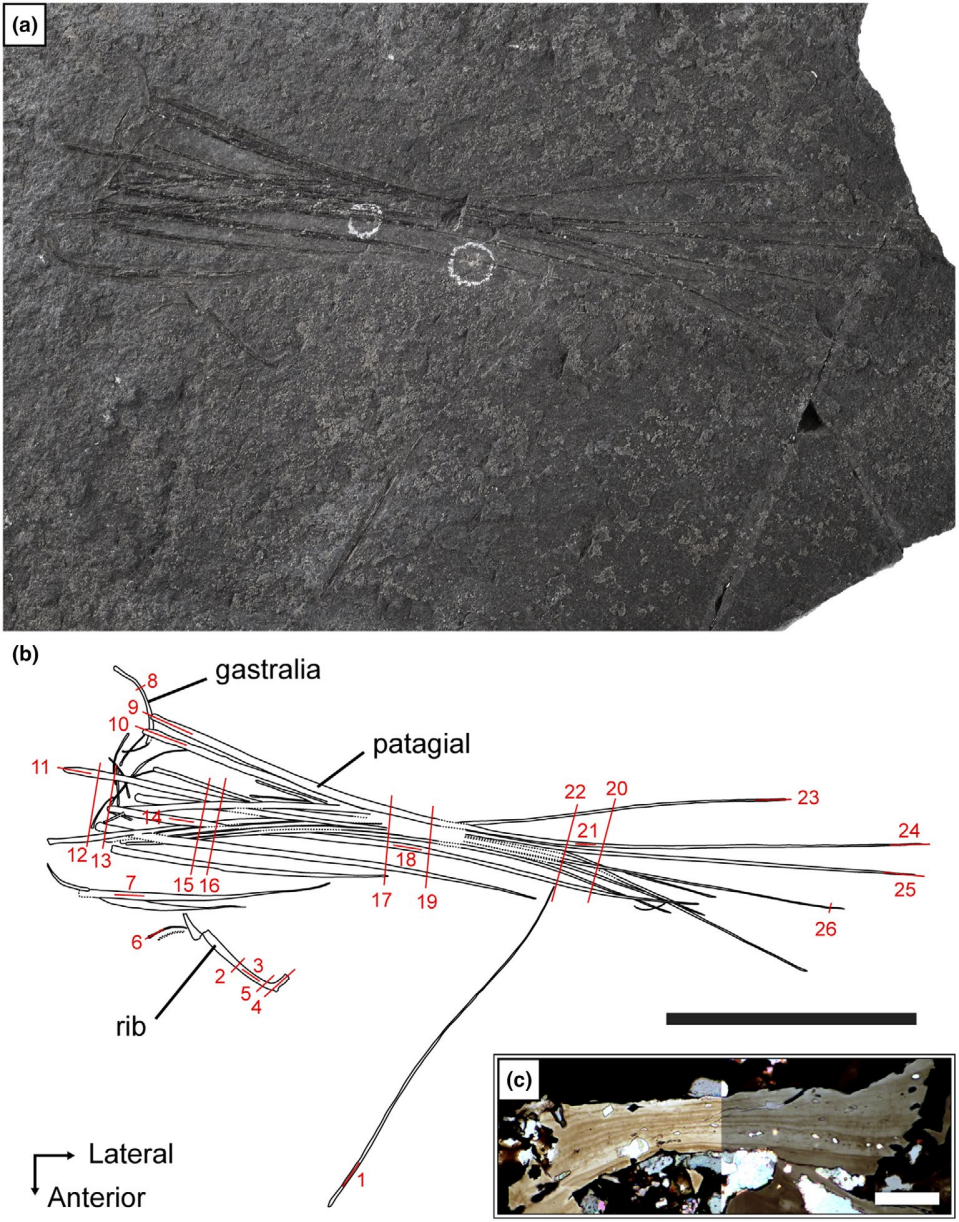
*Weigeltisaurus* sp. Kuhn, 1939 (Germany, Wuchiapingian), SMNK‐PAL 34865, partial right wing in lateral view. (a) Photograph and (b) line drawing. (c) Transverse section 2, fragment of rib cortex illustrating the little difference between normal light (left) and cross‐polarized light (right). Numbered red lines indicate the location and direction of the thin sections. Scale bar, 50 mm.

SMNK‐PAL 34865 represents a partial wing skeleton comprising ribs, gastralia and patagials in mostly anatomical position. The specimen was donated by a professional dealer and, because of the dark‐grey colored matrix, likely comes from a “Kupferschiefer” spoil heap of the Mansfeld or Bad Hersfeld area (Hese state, Germany) (Figure 2). However, the exact provenance of the specimen is unknown. The specimen consists of strongly compressed dark greyish‐black bones preserved on a finely laminated slab of slightly lighter dark grey shale.

In addition, fragments of patagials were extracted from SAM‐PK‐34865 and SAM‐PK‐34866b, the counterpart of the Bodental specimen. These fragments were mounted on small metal plates to allow for the observation of very fine anatomical details. The anatomical locations of these fragments are indicated on both specimens by a white circle drawn with a paint marker (Figure 2a).

Lastly, we also examined all specimens previously referred to *Coelurosauravus elivensis* (Buffa et al., 2021, 2022), as well as original specimens or high‐fidelity epoxy resin casts of most specimens previously referred to *Weigeltisaurus* (Evans, 1982; Evans & Haubold, 1987; Frey et al., 1997; Pritchard et al., 2021; Schaumberg, 1976, 1986; Schaumberg et al., 2007) for comparative purposes. The complete list of the studied material, following the taxonomic framework of Buffa et al. (2021, 2022), is provided in Table 1.

**TABLE 1:** Denominations and identifications of examined specimens referred to *Coelurosauravus* and *Weigeltisaurus*.

Denomination	Identification	Material examined	Remarks
SMNK‐PAL 34865	*Weigeltisaurus* sp.	SMNK‐PAL 34865	Specimen sampled for histological sections
Ellrich specimen or SMNK‐PAL 2882	*Weigeltisaurus* sp.	SMNK‐PAL 2882	Counterpart in private collection, unavailable for scientific study
*Weigeltisaurus* holotype or SSWG 113/7	*Weigeltisaurus jaekeli* (Holotype)	SMNK‐PAL 34899a (cast)	–
*Gracilisaurus* holotype or GM 1462	*Weigeltisaurus* sp.	SMNK‐PAL 34899b (cast)	–
Bodental specimen	*Weigeltisaurus* sp.	SMNK‐PAL 34866 (cast) & 34866b (original)	Main slab in Bürger private collection
Wolfsberg specimen	cf. *Weigeltisaurus*	SMNK‐PAL 34910 (cast)	Specimen in Naturkundemuseum im Ottoneum, Kassel
Bahaus specimen	*Weigeltisaurus* sp.	–	Specimen in Simon private collection
Eisleben specimen	cf. *Weigeltisaurus*	–	Specimen in Aue private collection
Brandt specimen	cf. *Weigeltisaurus*	–	Specimen in Brandt private collection
Eppelton specimen	*Weigeltisaurus* sp.	TWCMS B5937	–
MNHN.F.MAP325a	*Coelurosauravus elivensis* (Lectotype)	MNHN.F.MAP325a	Patrimonial number 1908‐11‐21a
MNHN.F.MAP317a & b	*Coelurosauravus elivensis* (Paralectotype)	MNHN.F.MAP317a‐b	Patrimonial number 1908‐11‐22a & b
MNHN.F.MAP327a & b	*Coelurosaravus elivensis*	MNHN.F.MAP327a‐b	Patrimonial number 1908‐5‐2

### 2.2 Imaging

Most weigeltisaurid specimens from Madagascar and Western Europe are exquisitely preserved and articulated, but their wings are often folded, making the identification of individual patagials difficult. We use reflectance transformation imaging (RTI) to generate “interactive specimens” on which the illumination can be oriented to help follow such superimposed, slender structures. We used the same custom‐made portable light dome as Buffa et al. (2021, 2022), an updated version of that presented by Béthoux et al. (2016), to capture sets of 54 photographs under different LED sources and compiled them using the RTI‐Builder software. Buffa et al. (2022) previously described RTI files of the patagium of *Coelurosauravus*, which were re‐analyzed herein, together with newly produced files for *Weigeltisaurus*. The resulting RTI files provided in Supplementary Data can be opened using the software RTIViewer (both software packages are freely available at www.culturalheritageimaging.org). In some cases, we extracted snapshots from the RTI files under the “normals visualization.” This setting displays the photographs in false colors, where the normal direction to each pixel is colored following a color map plotted on a hemisphere. This setting works best when examining thin, shallow imprints, such as the external molds left by gastralia in the matrix.

SMNK‐PAL 2882 was scanned using Computed Laminography (CL) at the Karlsruhe Institute of Technology in the Computed Laminography/Tomography Laboratory of the Institute for Photon Science and Synchrotron Radiation as described in Zuber et al. (2017). Two thousand and forty‐eight projections were acquired over a range of 360° of the tomography axis. Each projection was exposed for 2 × 4 s. The axis was inclined by 30° (laminographic angle). As source, an microfocus X‐ray tube (XWT‐225, X‐Ray WorkX GmbH Garbsen) was employed, operated with an acceleration voltage of 200 kV and a target power of 30 W. A flat panel detector was used (XRD 1621 CN14 ES, PerkinElmer, Waltham, USA). The cone‐beam magnification with the physical pixel size of 200 μm resulted in a voxel size of 6.91 μm. The 3D volume was reconstructed with the ufo/tofu framework (Faragó et al., 2022). To save storage space, the vertical slices showing only the matrix were removed. This dataset thus comprises 190 vertical slices with a resolution of 2046 × 2046 pixels.

Prior to sectioning, SMNK‐PAL 34865 comprised a thin rectangular slab of black shale (Figure 2). We did not scan the entire specimen because the very low thickness of the slab compared to its other dimensions may have caused reflection artifacts in the scans. Once sectioned, we selected one of the remaining fragments, embedded in epoxy resin, comprising the proximal parts of some patagials and adjacent gastralia for CT scanning at the Montpellier Ressources Imagerie Platform of the Institut des Sciences de l'Évolution—Montpellier, Université de Montpellier, France, using an EasyTom 150 (80 kV, 125 μA, voxel size 0.015 mm). The vertical slices were cropped to fit closely to the specimen, and slices showing only the matrix were removed. This dataset thus comprises 2579 vertical slices with a resolution of 188 × 1072 pixels.

For both SMNK‐PAL 2882 and SMNK‐PAL 34865, the vertical slices were imported into the 3D segmentation software Avizo 3D v.2021.1 (Thermo Fisher Scientific, Waltham, MA). Three‐dimensional STL surface models of ribs, gastralia, and patagials were generated in individual “labels” and “materials” using the paint brush tool with given gray value thresholds.

### 2.3 Thin sectioning

Specimen SMNK‐PAL 34865, comprising a partial wing including ribs, gastralia, and patagials of *Weigeltisaurus* sp., was sampled for histological analysis in the thin section lab of the MNHN (Figure 2). The specimen was first cut into smaller pieces around the areas selected for transversal and longitudinal histological sampling. Each piece was then embedded in epoxy resin before being thin‐sectioned and polished. In total, 26 thin sections were made from this specimen, including transverse and longitudinal sections of a single rib, a few gastralia, and of the patagials at different regions along their length. The study of both transverse and longitudinal sections is indeed required to get an understanding of the three‐dimensional structure of the bone tissue (Stein & Prondvai, 2014). The locations of the 26 thin sections produced from SMNK‐PAL 34865 are illustrated in Figure 2b. These thin sections, as well as a high‐fidelity epoxy resin cast of the specimen made before sectioning, are housed in the paleontology collection of the SMNK.

The thin sections of SMNK‐PAL 34865 were observed under normal transmitted light and under cross‐polarized light. However, the latter did not show any structure (Figure 2c). This is probably due to the compact cortex of parallel‐fibered bone (described below) of the sampled elements, which do not show any birefringence pattern. As a result, the histological sections presented here are only described under normal transmitted light. The histological nomenclature used below follows de Buffrénil et al. (2021).

### 2.4 Geometric and microanatomical parameters

Two thin sections of a gastralium were binarized by hand drawing the sections in black over a white background for subsequent analysis in R using the BoneProfileR package (Gônet et al., 2022), which is an enhanced version of the Bone Profiler software (Girondot & Laurin, 2003). BoneProfileR is designed to quantitatively analyze bone microanatomy in cross section through the modeling of compactness profiles. Foreground/background colors and bone centers were detected automatically using the dedicated functions in the package. We used the ontogenetic center to estimate bone compactness. The ontogenetic center is the putative center from which the bone developed. The bone section is divided into 60 sections of 6° from the center to the periphery and on 100 concentric slices (default values). The compactness is estimated for each of these 6000 portions of the bone section. The compactness profile from center to periphery is modeled using a scaled logit or flexit (flexible‐logit) function based on a binomial distribution (mineralized or non‐mineralized pixel). We followed a three‐step procedure to fit the two functions: first, a quasi‐Newton method based on maximum likelihood; then, a Bayesian Markov chain Monte Carlo approach to escape potential local optima and to obtain distributions of parameters; and again, a quasi‐Newton method to improve the probability that the maximum likelihood is reached. The scaled logit and flexit models were compared using the Akaike information criterion to limit over‐parametrization. We then performed radial analyses for each of the 6° sections to assess the variation of cortical thickness. Observed compactness (bone to unmineralized pixel ratio) was extracted from the analysis reports and subsequently plotted to facilitate biological interpretation.

None of the patagial sections show a complete and undeformed patagial cross section. Furthermore, the biconcave outline of the cross section of the patagials hampered the bone center detection steps in BoneProfileR, which did not manage to follow the outline of the preserved sections. Each studied section thus had to be further divided into three to five subsections targeted at the best‐preserved regions of the bone, which could be successfully analyzed by BoneProfileR. To this aim, we estimated the position of the center of the section based on the bi‐axial symmetry of the patagial cross sections and divided the section into three to five parts, which were analyzed individually with manual centering. The subsections were then processed following the same analytical workflow as the complete sections (see above).

### 2.5 Institutional abbreviations

GM, Geiseltalmuseum, Martin‐Luther‐Universität, Halle, Germany; LSUMZ, Louisiana State Museum of Natural History; MNHN, Muséum national d'Histoire naturelle, Paris, France; SMNK, Staatliches Museum für Naturkunde Karlsruhe, Karlsruhe, Germany; SSWG, Sektion Geologie, Ernst‐Moritz‐Arndt Universität, Greifswald, Germany; TWCMS, Sunderland Museum, Tyne and Wear County Museums, Sunderland, United Kingdom.

## 3 RESULTS

Here, we provide the first anatomical description of SMNK‐PAL 34865 prior to sectioning. Then, we conduct a comparative anatomical examination of the gastral basket and patagial skeleton of the best preserved weigeltisaurid specimens from Germany and Madagascar. This is mostly based on the new information brought to light by the RTI and CL examination of the Ellrich specimen SMNK‐PAL 2882. A full redescription of the specimen based on its CL scan is beyond the scope of this study and is currently being undertaken by the authors. Lastly, we qualitatively and quantitatively describe the histology and microanatomy of SMNK‐PAL 34865 based on the newly produced thin sections.

### 3.1 Description and identification of SMNK‐PAL 34865

**FIGURE 3:**
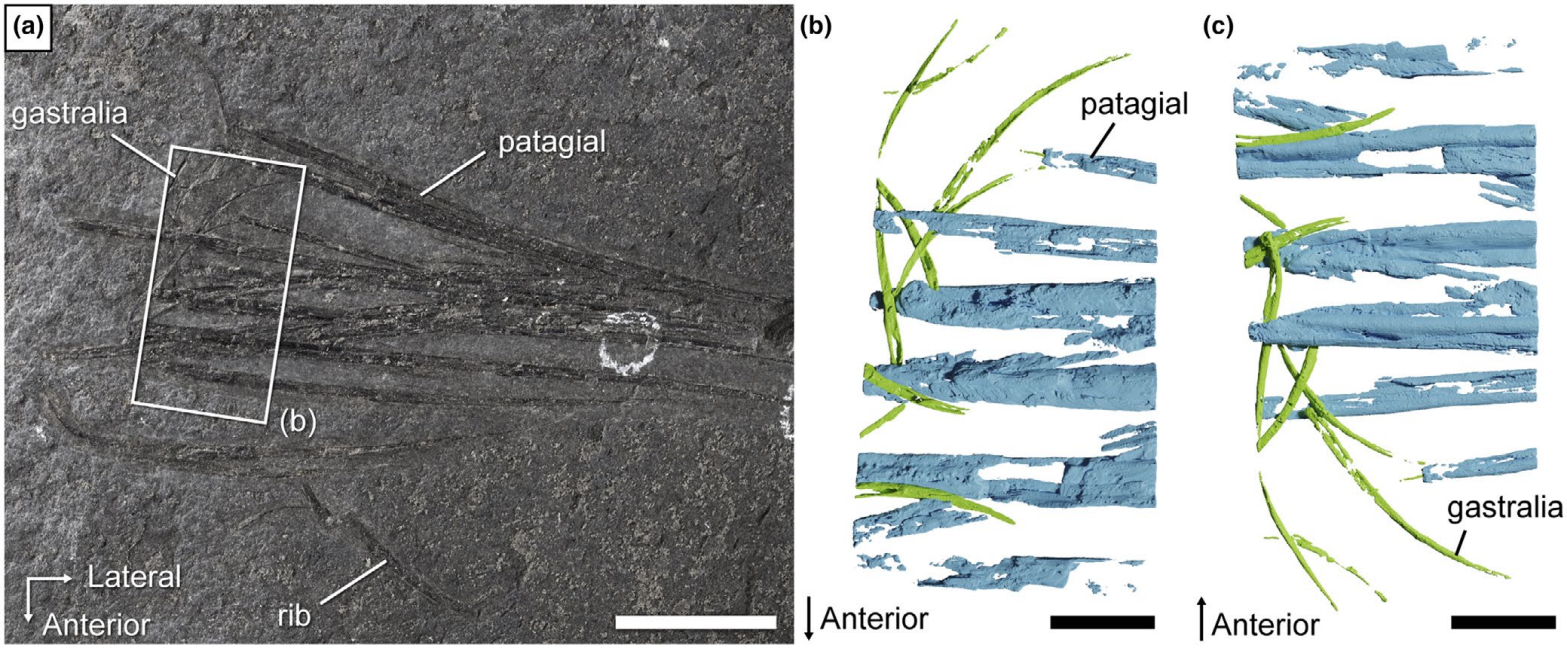
*Weigeltisaurus* sp. Kuhn, 1939 (Germany, Wuchiapingian), SMNK‐PAL 34865, close‐up of the base of the patagial skeleton in mostly dorsal view (a) and surface model of the segmented gastralia and patagial from a fragment of the specimen after sectioning in dorsal (b) and ventral views (c). Scale bar, 20 mm (a), 5 mm (b, c).

SMNK‐PAL 34865 shows at least two ribs, 15 gastralia, and 15 patagials (Figure 2). The exposed surfaces of the bones are mostly missing, presumably as a result of the splitting of the slab into part and counterpart. The latter was not recovered from the spoil heap. The ribs are located anterior to the rest of the preserved skeletal elements and are much more offset from the base of the patagials than the ribs preserved in *Coelurosauravus* (MNHN.F.MAP327a), suggesting that they were subject to some postmortem displacement. One of the ribs is only represented by a fragment, but the other one is complete. As preserved, this rib is dichocephalous and strongly L‐shaped, with the capitulum extending at a roughly right angle to the shaft (Figure 3a). The rib shaft is only slightly curved and subtly thickens ventrally. The morphology of this rib conforms well to the anterior and middle dorsal ribs of *Coelurosauravus* (MNHN.F.MAP327a) and is also reminiscent of the more poorly preserved anterior dorsal ribs of *Weigeltisaurus* (SMNK‐PAL 2882). Both ribs of SMNK‐PAL 34865 are thus likely anterior dorsal ribs.

The gastralia are mostly located near the base of the patagials except for a displaced element that lies near the distal tip of the patagials (Figures 2 and 3). The gastralia are thin crescentic bones, similar to those of *Weigeltisaurus* (SMNK‐PAL 2882). Their lateral terminus is expanded into a small bulbous head that lies in close association with the patagials while the medial end tapers to a point, as described for *Coelurosauravus* (Buffa et al., 2022). As preserved in SMNK‐PAL 34865, the gastralia appear to show a preferential anteroposterior orientation with the thick lateral head lying anteriorly in close association with the patagials (Figure 3). However, this may not reflect the true orientation of these bones as the trunk has collapsed due to postmortem compression. Lastly, the gastralia of SMNK‐PAL 34865 do not seem to be connected to one another, similar to the condition in *Weigeltisaurus* (SMNK‐PAL 2882), where the gastral basket is formed by only one gastralium on each side.

The patagials are partially superimposed, precluding a definite count and making some of them impossible to trace throughout their length (Figure 2). One patagial underwent strong postmortem displacement and lies anterior to the rest of the wing. The proximal ends of the patagials appear to mostly overlap the gastralia, which would indicate that the specimen represents part of a right wing, as the patagials lie lateral to the gastralia in both *Coelurosauravus* (MNHN.F.MAP327a) and *Weigeltisaurus* (SMNK‐PAL 2882). However, some of the underlying patagials probably pertain to the left wing as well, as the trunk of the animal collapsed during postmortem decay and compression when the carcass reached the seafloor. The patagials of SMNK‐PAL 34865 do not appear to form bundles as they do in the Ellrich specimen of *Weigeltisaurus* SMNK‐PAL 2882 (Frey et al., 1997; Pritchard et al., 2021) but instead seem to be rather regularly spaced (Figures 2 and 3) as seen in *Coelurosauravus* (MNHN.F.MAP327a).

The anterior‐most four patagials are nearly complete and can be confidently followed (Figure 2). They measure ca. 29.76, 50.11, 55.45, and 89.12 mm in chord length, respectively (i.e., in a straight line from the proximal to the distal end, as in Buffa et al., 2022). This rapid increase in length conforms well with that of the anterior‐most patagials of *Coelurosauravus* (MNHN.F.MAP327a). The most posterior preserved patagials can also be confidently traced and represent the longest preserved patagials, reaching chord lengths of 156.15 and 157.17 mm, respectively. By comparison with *Coelurosauravus*, in which the patagial length increases rapidly in the anterior third of the wing before gradually decreasing (Buffa et al., 2022), we suggest that SMNK‐PAL 34865 preserves the anterior portion of a right wing, likely overlying the anterior part of the left wing (Figure 2).

SMNK‐PAL 34865 thus represents a partial weigeltisaurid anterior wing skeleton (Figures 2 and 3). Only one character to distinguish weigeltisaurid genera has been proposed on this portion of the skeleton: the organization of the patagials (Buffa et al., 2022). The patagials are indeed regularly spaced in *Coelurosauravus* (Buffa et al., 2022) but clearly form bundles in the Ellrich specimen (Figure 1; Frey et al., 1997; Pritchard et al., 2021; Schaumberg et al., 2007). We tentatively identify bundles in the holotype of *Glaurung* as well (Bulanov & Sennikov, 2015; Schaumberg et al., 2007), although the preservation of the material (not directly studied by us) precludes a definite statement. In SMNK‐PAL 34865, the patagials do not form any clear bundles (Figures 2 and 3), thus being more similar to those of *Coelurosauravus* than to those of the Ellrich specimen. However, the patagials are also regularly spaced in the Bodental (SMNK‐PAL 34866b; Schaumberg, 1986), Bahaus (Schaumberg, 1976), and Eppelton (TWCMS B5937; Evans, 1982) specimens, which are currently referred to the genus *Weigeltisaurus*.

Given the present delimitation of the genus *Weigeltisaurus* (Table 1), it is thus impossible to unequivocally refer SMNK‐PAL 34865 to *Weigeltisaurus* on anatomical grounds. Similarly, a *Rautiania* identification cannot be excluded because none of the bones preserved in SMNK‐PAL 34865 (ribs, gastralia, patagials) have been reported in the Russian weigeltisaurids (Bulanov & Sennikov, 2006, 2010). However, based on circumstantial evidence—*Rautiania* currently being known only from Eastern European deposits (Bulanov & Sennikov, 2010)—we here refer SMNK‐PAL 34865 to *Weigeltisaurus* sp. with a reasonable degree of confidence, pending a revision of the alpha taxonomy of the genus *Weigeltisaurus*.

### 3.2 Comparative anatomy of the weigeltisaurid gastral basket

Although the gastral basket is reasonably well preserved in several weigeltisaurid specimens, it represents the least studied region of the skeleton. Pritchard et al. (2021) and Buffa et al. (2022) were the first to describe these structures in detail in the Ellrich specimen of *Weigeltisaurus* and in *Coelurosauravus*, respectively. Both studies highlighted a close association between gastralia and patagials, which prompted a detailed reassessment of the gastral basket in weigeltisaurids.

**FIGURE 4:**
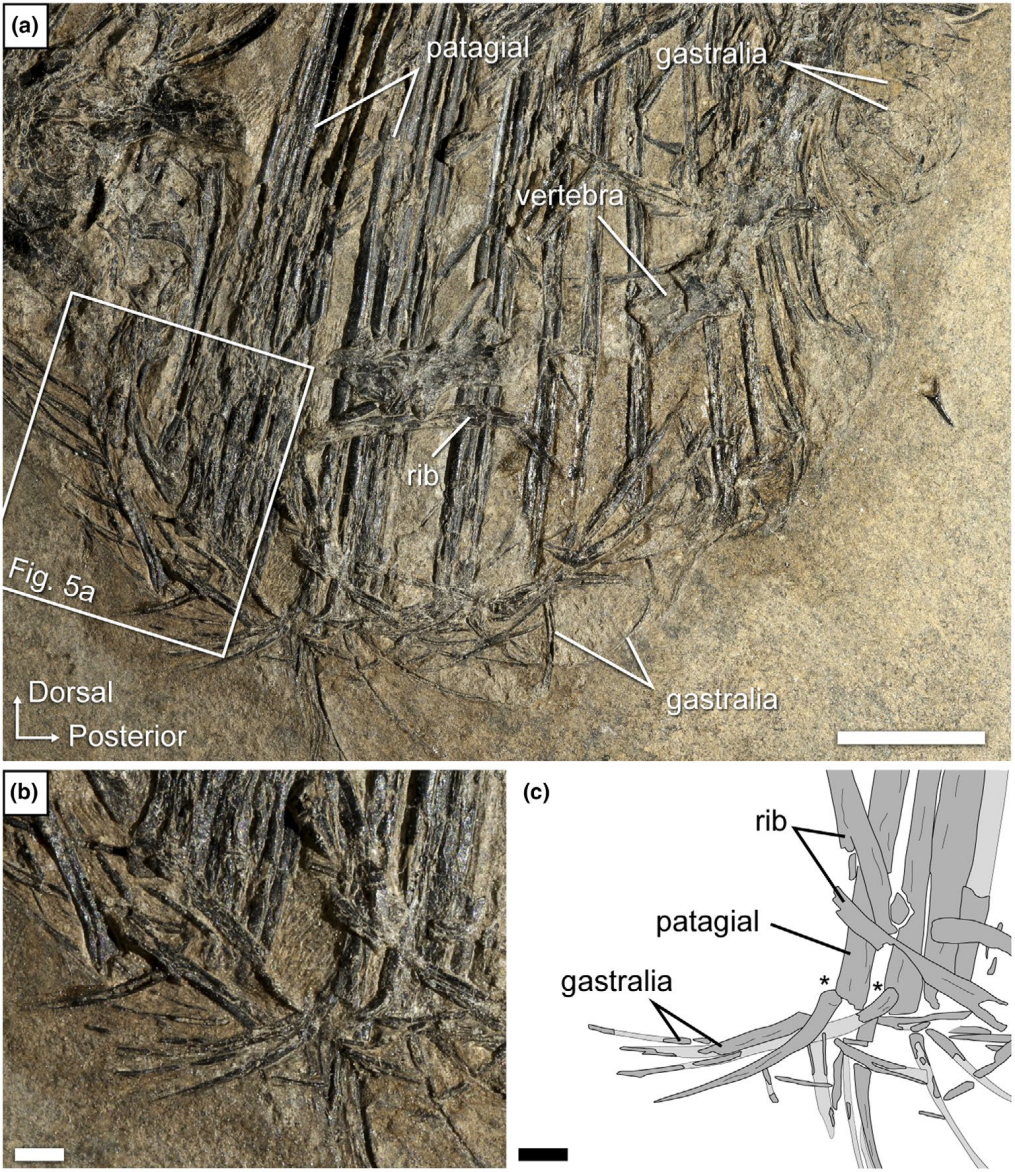
*Weigeltisaurus* sp. Kuhn, 1939 (Germany, Wuchiapingian), Ellrich specimen SMNK‐PAL 2882. (a) Photograph of the gastral basket and the proximal portion of the patagials. The trunk is overall in left lateral view but the extended right wing is visible in dorsal view. Close‐up (b) and interpretive drawing (c) of the base of the second bundle of patagials. “*” indicates very close association between a gastralium and a patagial. Scale bars, 10 mm (a), 2 mm (b, c).

**FIGURE 5:**
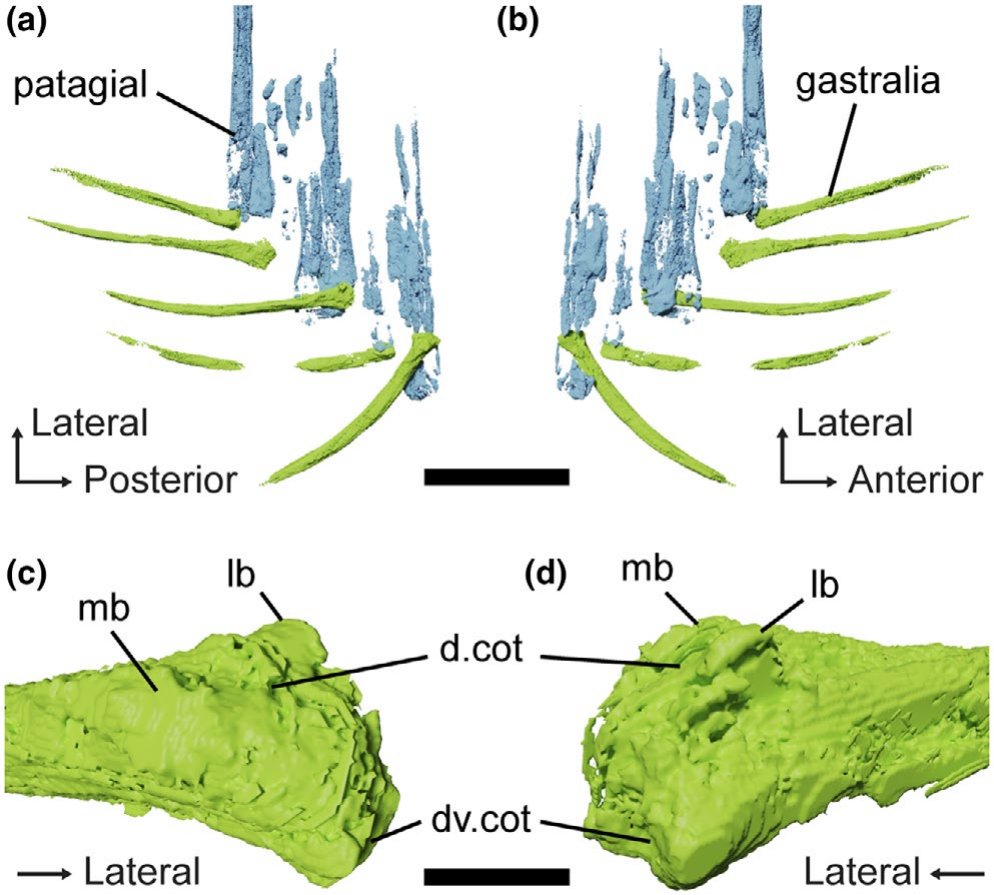
*Weigeltisaurus* sp. Kuhn, 1939 (Germany, Wuchiapingian), Ellrich specimen SMNK‐PAL 2882, surface models of the anterior trunk showing the gastralia and base of patagials in mostly dorsal (a) and ventral (b) views, highlighting the very close association between these elements. Close‐up of the lateral extremity of a gastralium in oblique posterolateral (c) and anterolateral (d) views. d.cot, dorsal cotyle; dv.cot, distoventral cotyle; lb., lateral boss; mb, medial boss. Scale bars, 5 mm (a, b), 500 μm (c, d).

The Ellrich specimen SMNK‐PAL 2882 preserves a nearly complete gastral basket, which will serve as the main basis for the comparative description of this structure in weigeltisaurids. This specimen is visible in mostly left lateral view, but the right wing is fully extended and exposed in dorsal view, being overlain by the gastralia and vertebral fragments (Figure 1). Parts of the folded left wing overly the right patagials and trunk. Presumably, the patagial skeleton of the left wing is preserved on the counterpart slab. The specimen has undergone postmortem compression, which resulted in the collapse of the thorax, and to a lesser extent of the abdomen, in such a way that individual gastral rows are hard to identify (Figure 4). However, the CL examination of this specimen allows for a better examination of this part of the skeleton (Figure 5).

The gastralia of SMNK‐PAL 2882 are thin splint‐like bones that lie level with the ventral margin of the animal (Figure 4a). They appear to cover most of the trunk length. However, as the pectoral girdle is poorly preserved and slightly displaced due to postmortem compression, it remains unclear whether the gastral basket began immediately posterior to the coracoid plate. In *Coelurosauravus*, for which a well‐preserved trunk skeleton is known, there is a gap between the first gastral row and the posterior margin of the coracoids. This gap has been interpreted as the location of a cartilaginous sternum (Buffa et al., 2022; Carroll, 1978).

**FIGURE 6:**
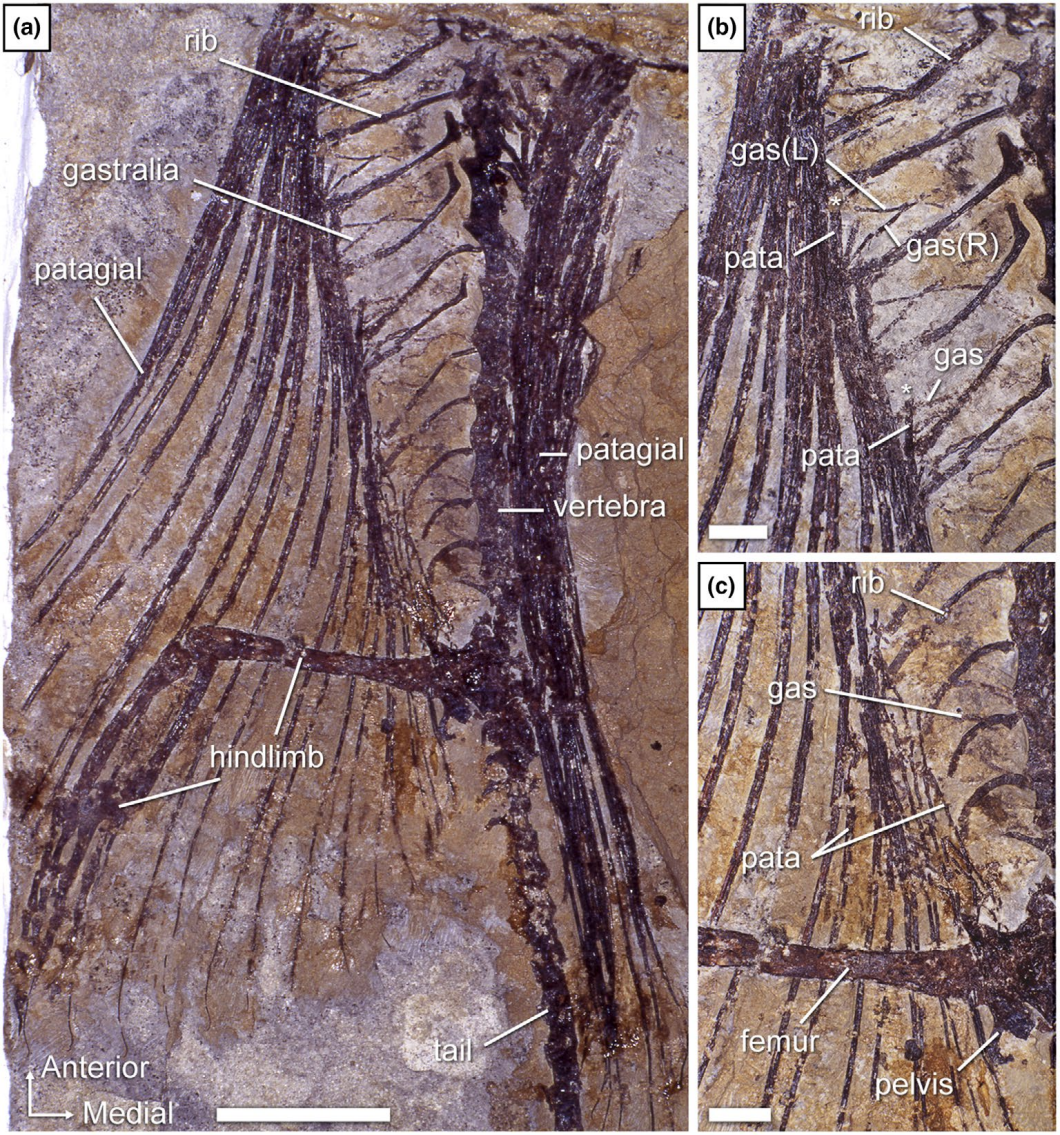
*Weigeltisaurus* sp. Kuhn, 1939 (England, Wuchiapingian), Eppelton specimen TWCMS B5937. Photographs of the entire wing region (a) and close‐ups of the anterior (b) and posterior (c) portions of the preserved gastral basket and the base of the associated patagials. “*” indicates a very close association between a gastralium and a patagial. Gas, gastralia; pata, patagium. Scale bars, 20 mm (a), 5 mm (b, c).

**FIGURE 7:**
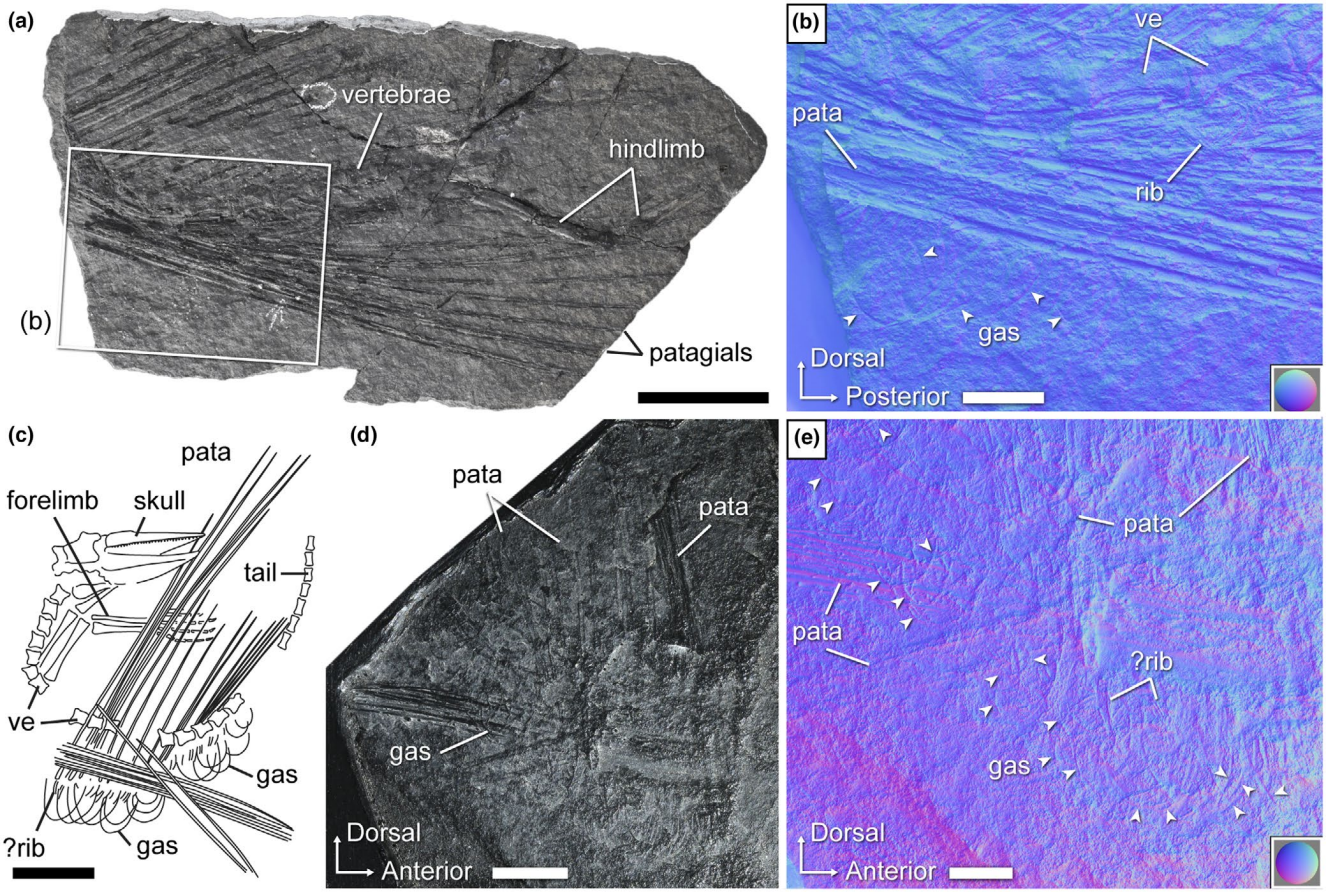
*Weigeltisaurus* sp. Kuhn, 1939 (Germany, Wuchiapingian). (a, b) Counterpart of Bodental specimen SMNK‐PAL 34866b. Photograph (a) and close‐up of the anterior trunk under “normals visualization” (extracted from RTI file, see Methods) (b). (c–e) Wolfsberg specimen, main part (privately owned) and SMNK cast of counterpart SMNK‐PAL 34910. (c) Interpretive drawing of main part (not studied by us, redrawn from Schaumberg, 1986). Photograph (d) and close‐up of the trunk under “normals visualization” (extracted from RTI file, see Methods) (e). Color circles in the bottom left corner of (b, e) indicate color code for normal directions as applied to a hemisphere. Triangular arrows point to the gastralia. Gas, gastralia; pata, patagium; ve, vertebra. Scale bars: 20 mm (a), 10 mm (c, d), 5 mm (b, e).

**FIGURE 8:**
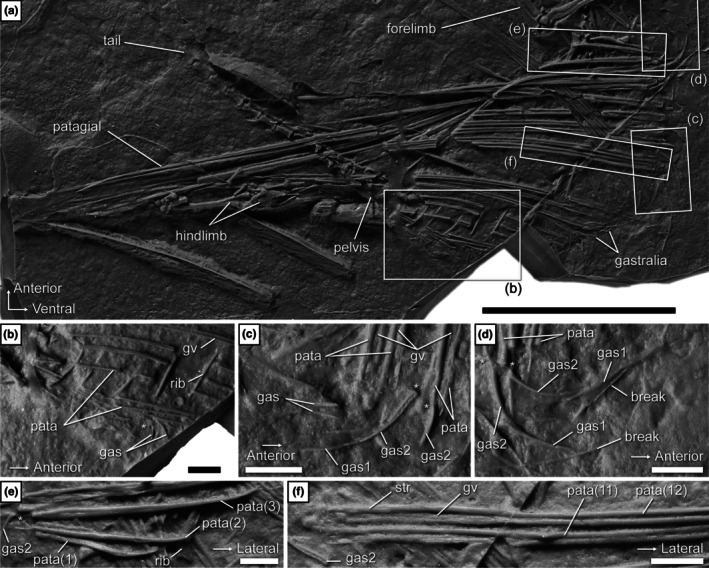
*Coelurosauravus elivensis* Piveteau, 1926 (Madagascar, Late Permian) MNHN.F.MAP327a, silicone cast of individual preserved as an external mold. Photograph of the entire wing region (a) and close‐ups of the posterior (b), middle (c), and anterior (d) portions of the gastral basket, the base of the associated patagials, and of patagials 1–3 (e) and 11 and 12 (f). “*” indicates very close association between a gastralium and a patagial. gas1, medial gastralium; gas2, lateral gastralium; gv, groove; pata, patagial (numbers indicate position along wing); str, striations. Scale bars, 50 mm (a), 5 mm (b‐f).

Although the individual gastral transverse rows (hereafter simply “rows”) of SMNK‐PAL 2882 were slightly disarticulated when the trunk collapsed, the gastralia do not appear to articulate with each other (Figures 4 and 5). Each gastral row thus comprises only one element on either side. A similar morphology occurs in the Eppelton specimen of *Weigeltisaurus*, which is mostly preserved in dorsoventral view (Figure 6a). In this specimen, the midline of the gastral rows lies just left of the vertebral column. The gastral rows have collapsed due to postmortem compression so that the single element from each side intersects, forming an X‐shaped pattern along the midline of the trunk (Figure 6b). Similarly, the RTI examination of the Bodental and Wolfsberg specimens, both of which preserve the gastral rows in lateral view, did not show evidence of more than one gastralium on either side (Figure 7). These specimens thus likely had single‐element gastral rows as well, although the preservation of the material precludes a definite statement. In contrast, in *Coelurosauravus*, each gastral row comprises two elements on either side, the lateral one (gastralium 2) being significantly more curved than the medial one (gastralium 1) (Figure 8c,d).

The more anterior gastralia of SMNK‐PAL 2882 are relatively long and slender bones compared to the more posterior ones, showing only a moderate degree of curvature (Figures 4b,c and 5a), which conforms to those of SMNK‐PAL 34865, as best seen on the segmented surface models (Figure 3b,c). Our RTI examination of SMNK‐PAL 2882 reveals that, in the best‐preserved elements, one end of each gastralium is closely associated with the underlying base of a patagial (Figure 4c). This is even clearer on the surface models (Figure 5a). In all cases, the end of the gastralium associated with a patagial is enlarged and knob‐like while the one pointing away from the patagial tapers to a point. By comparison with the lateral gastralia of the better preserved gastral rows of *Coelurosauravus* (Figure 8c,d), we identify the tapering end of the gastralia of SMNK‐PAL 2882 as the medial one and the knob‐like end as the lateral one.

SMNK‐PAL 2882 shows a complex morphology of the lateral end of the gastralia (Figure 5b,c). In general outline, the lateral ends of the gastralia are expanded relative to the mid‐shaft by about one‐third and show a slight ventral curvature. This termination bears two concavities resembling articular cotyles, one on the dorsal surface and one on the distoventral surface (Figure 5b,c). Of these, the dorsal cotyle is framed by medial and lateral bosses. In light of this complex surface and the close association between the lateral end of the gastralia and the base of the patagials in all weigeltisaurid specimens, both structures were likely articulated with each other, possibly through a double condyle‐cotyle joint. However, this interpretation is hampered by the incomplete preservation of the proximal ends of the associated patagials in SMNK‐PAL 2882.

In contrast to the anterior ones, the more posterior gastralia of SMNK‐PAL 2882 are more strongly curved and semicircular, which gives the posterior trunk a nearly cylindrical outline as preserved (Figure 4a). Similarly curved posterior gastralia are also seen in the Wolfsberg specimen of *Weigeltisaurus* (Figure 7c–e), as well as in *Coelurosauravus*, although the posterior gastralia are mostly obscured by the patagials in MNHN.F.MAP327a, the only specimen to preserve this part of the skeleton (Figure 8b).

The trunk of SMNK‐PAL 2882 has collapsed, so the orientation of the gastral rows is uncertain. As preserved, the anterior‐most gastralia appear posterolaterally oriented, with their lateral ends lying posterior to the medial ones. This suggests that the anterior gastral rows are organized in a series of anteriorly oriented chevrons spanning the entire width of the anterior trunk. The gastralia of the mid‐trunk do not appear to show any preferential orientation, with the medial ends pointing either anteriorly, ventrally, or posteriorly with respect to the lateral one (Figure 4a). As described above, the posterior gastral rows appear mostly in anatomical position, forming ventrally vaulted semi‐circular rows. However, the orientation of the gastral rows relative to the coronal plane remains unclear. As described by Buffa et al. (2022), the *Coelurosauravus* specimen MNHN.F.MAP327a shows a similar variation in the orientation of the gastralia as preserved along the trunk length (Figure 8b–d). In contrast, the gastral rows of the Bodental and Wolfsberg specimens of *Weigeltisaurus* show a semicircular pattern for all preserved gastral rows that is reminiscent of the posterior gastral rows of SMNK‐PAL 2882 (Figure 7b,c,e). This pattern thus occurs more anteriorly than preserved in SMNK‐PAL 2882 because the Bodental specimen preserves anterior to mid‐trunk gastral rows while the Wolfsberg specimen preserves a nearly complete series. However, both specimens are missing the anterior gastral rows. Lastly, the mostly dorsoventrally preserved Eppelton specimen shows nearly transversely oriented gastral rows throughout its preserved trunk portion, each gastralium showing only a slight posterolateral orientation. However, this specimen is missing the anterior part of the trunk (Figure 6b,c).

**FIGURE 9:**
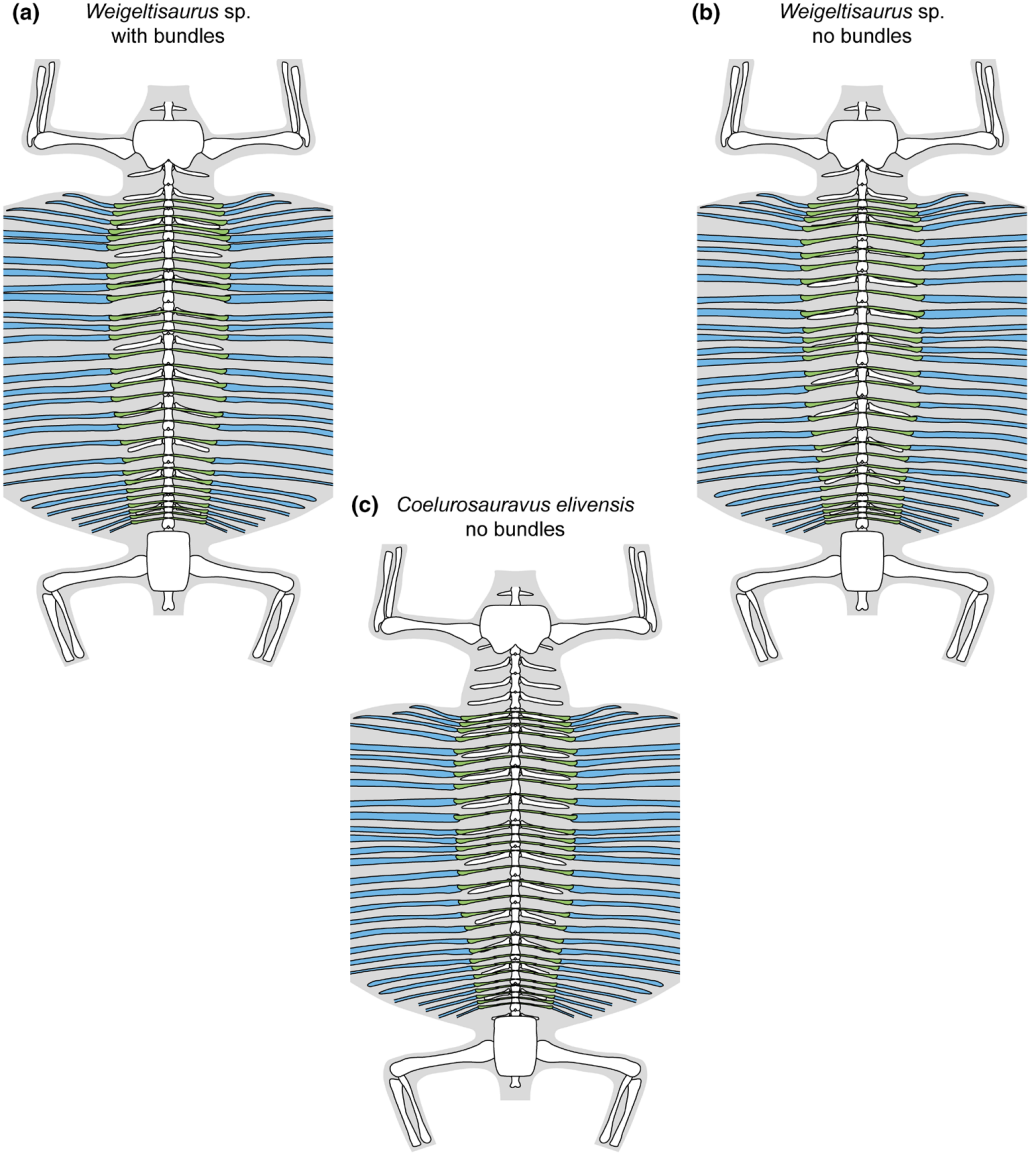
Reconstruction of the gastral (green) and patagial (blue) skeleton in weigeltisaurids. (a) *Weigeltisaurus* sp. Kuhn, 1939, bundled morphotype based on SMNK‐PAL 2882 (modified in part from Pritchard et al., 2021). (b) *Weigeltisaurus* sp. Kuhn, 1939, non‐bundled morphotype based primarily on TWCMS B5937. (c) *Coelurosauravus elivensis* Piveteau, 1926 (modified from Buffa et al., 2022). Reconstructions not to scale.

The ventrally vaulted semicircular pattern of the gastral rows visible on the Bodental and Wolfsberg specimens conforms well to the transverse X‐shaped pattern preserved in the Eppelton specimen, which allows for the first reconstruction of the pattern of gastral rows in weigeltisaurids (Figure 9). We suggest that, in all specimens of *Weigeltisaurus*, the gastral rows show only a slight posterolateral orientation, forming obtuse anteriorly oriented chevrons in which the gastralia are mostly oriented transversely, extending from the sagittal plane to the base of the patagials. These gastral rows would yield a transverse X‐shaped pattern when the trunk collapses dorsoventrally, as seen in the Eppelton specimen (Figure 6), or a ventrally vaulted semicircular pattern when the trunk collapses transversely, as seen in the Bodental and Wolfsberg specimens (Figure 8). However, if the trunk is strongly coiled, this may result in the loss of the gastral row orientation, as is the case in the Ellrich specimen (Figure 3). Due to the similarities in preservation between the Ellrich specimen of *Weigeltisaurus* and the MNHN.F.MAP327a of *Coelurosauravus*, we suggest that both taxa had a similar orientation of the gastral rows.

### 3.3 Comparative anatomy of the weigeltisaurid patagial skeleton

#### 3.3.1 Patagial count and overall shapes

We concur with Pritchard et al. (2021) who identified at least 24 patagials on the right wing of the Ellrich specimen (Figure 1), slightly more than the minimum of 22 patagials reported by Frey et al. (1997). However, the short and thin posterior patagials are hard to identify, so the exact count may be slightly higher. We also report a minimum count of 29 patagials in specimen MNHN.F.MAP327a of *Coelurosauravus* (Figure 8a), in agreement with previous studies (Buffa et al., 2022; Pritchard et al., 2021). Among sub‐complete specimens, we report a minimal count of 24 patagials on the left wing of the Eppelton specimen, slightly more than the 21 “distal ribs” reported by Evans (1982) and the 23 patagials of Pritchard et al. (2021). This specimen preserves an anterior patagial that is markedly shorter than the others, lying in second position in the left wing as preserved (Figure 6a). By comparison with the more complete anterior wings of other *Weigeltisaurus* specimens (SMNK‐PAL 2882, SMNK‐PAL 34865; Figures 1, 2, 3) and of *Coelurosauravus* (MNHN.F.MAP327a), we suggest that at least three to four patagials may be missing in the Eppelton specimen, adding up to a minimum count of 27 or 28 patagials. This conforms to previous estimates (Evans, 1982; Pritchard et al., 2021). In addition, Schaumberg (1976) reported a minimum of 23 patagials in the Bahaus specimen and 15 in the Wolfsberg specimen, and we here report 16 patagials on the left wing of the counterpart of the Bodental specimen (Figure 7a), all of which are incomplete anteriorly. In summary, based on all available specimens, we concur with Pritchard et al. (2021) that all weigeltisaurids likely had between 25 and 30 patagials in each wing.

In SMNK‐PAL 2882, the patagials are arranged in bundles (Figures 1 and 4; Frey et al., 1997; Pritchard et al., 2021; Schaumberg et al., 2007). In contrast, the exquisitely preserved MNHN.F.MAP327a specimen of *Coelurosauravus* shows roughly regularly spaced patagials (Figure 8a). Buffa et al. (2022) considered the regular patagial organization of *Coelurosauravus* as a diagnostic character of the genus by comparison with SMNK‐PAL 2882, the best‐known specimen currently attributed to *Weigeltisaurus*. However, our detailed examination of most specimens referred to *Weigeltisaurus* shows that the patagials are also regularly spaced in the Eppelton (Figure 6a), Bodental (Figure 7a), and Bahaus (Schaumberg, 1976) specimens. We tentatively identify bundled patagials in the counterpart of the Wolfsberg specimen (Figure 7d) and in the holotype and only known specimen of *Glaurung* (Bulanov & Sennikov, 2015; Schaumberg et al., 2007). Aue (2001, p. 14) reported “partial bundling” in the Eisleben specimen as well. However, we refrain from making a definite statement as we did not examine all of the relevant material and due to their preservation. None of the other known weigeltisaurid specimens are sufficiently well preserved to assess the organization of the patagials, including the *Weigeltisaurus* holotype.

Specimen MNHN.F.MAP327a of *Coelurosauravus* and SMNK‐PAL 2882 of *Weigeltisaurus* are the only two weigeltisaurid specimens in which the length of most of the patagials can be measured (Table 2). Both specimens show a very quick increase in length in the first patagials, so that the longest fully preserved patagials of the wing correspond to patagial 9 in MNHN.F.MAP327a and patagial 8 in SMNK‐PAL 2882 (Table 2). Considering the incompleteness of the following patagials (especially in SMNK‐PAL 2882), it appears that the wing reaches is maximum span around the level of the 10th patagial in all weigeltisaurids, roughly corresponding to the anterior third of the anteroposterior length of the wing. Buffa et al. (2022) reported a gradual decrease in patagial length from patagials 10–14 in MNHN.F.MAP327a, followed by a more rapid decrease more posteriorly. Although most of the middle and posterior patagials of SMNK‐PAL 2882 are incomplete, what is preserved conforms to the morphology of MNHN.F.MAP327a (Table 2; Figure 1).

**TABLE 2:** Patagial length in *Coelurosauravus elivensis* Piveteau, 1926 (taken from Buffa et al., 2022) and *Weigeltisaurus* cf. *jaekeli* (Weigelt, 1930).

Patagial	MNHN.F.MAP327a (mm)	SMNK‐PAL 2882 (mm)
Patagial 1	15.47	16.03
Patagial 2	19.41	31.38
Patagial 3	28.81	>44.69
Patagial 4	41.74	80.67*
Patagial 5	60.71	118.58*
Patagial 6	86.20	144.91
Patagial 7	105.76*	152.83
Patagial 8	152.32	166.02
Patagial 9	162.84*	>104,03
Patagial 10	>53.32	>121.51
Patagial 11	118.21*	>123.21
Patagial 12	117.56*	>41.04
Patagial 13	129.84*	>89.50
Patagial 14	133.74*	>90.82
Patagial 15	>16.53	>125.01
Patagial 16	130.11*	>30.69
Patagial 17	>56.36	92.98
Patagial 18	>49.56	>70.10
Patagial 19	73.20	Incomplete
Patagial 20	>39.66	Incomplete
Patagial 21	>37.22	Incomplete
Patagial 22	>50.15	Incomplete
Patagial 23	>12.95	Incomplete
Patagial 24	>12.93	Incomplete
Patagial 25	>11.15	–
Patagial 26	>10.97	–
Patagial 27	>11.60	–
Patagial 28	>15.709	–
Patagial 29	>6.162	–

*Note*: “*” indicates approximate measurements due to incomplete preservation. Length measured along the line linking the medial and lateral extremities of each patagial (“chord length” of Buffa et al., 2022), ignoring the curvature of each element.

As seen in SMNK‐PAL 2882 and MNHN.F.MAP327a, the individual patagials are long and thin rods that maintain a roughly constant width for at least two‐thirds of their length before gradually tapering distally (Figures 1 and 8a). As preserved, the patagials of these two specimens show a slight anteriorly convex curvature at their midline that spans roughly the middle third of their length. This conforms well to the curvature of the spars seen in dorsal view in the Eppelton specimen (Figure 6a). Each spar maintains a roughly constant width for at least two‐thirds of its length before tapering distally. As seen in SMNK‐PAL 2882 and MNHN.F.MAP327a, patagials 1–10 gradually thicken, whereas the following ones become progressively thinner (Figures 1 and 8a).

As described by Buffa et al. (2022) for MNHN.F.MAP327a, patagial 1 is sigmoidal in *Coelurosauravus*, curving anteriorly and then posteriorly in its distal half (Figure 8e). A similar morphology is seen in SMNK‐PAL 34865 despite its less pristine preservation (Figures 2 and 3a). Owing to the latter specimen, we suggest that the first patagial of SMNK‐PAL 2882, which was considered mostly straight by Pritchard et al. (2021), may have also been sigmoidal, but this shape could have been obliterated by postmortem compression. In this context, we also remain cautious regarding the interpretation of distal curvature of the patagials in SMNK‐PAL 2882, in which at least patagials 4–6 show a slight distal posterior curvature (Figure 1). A similar curvature is also seen in patagials 2–4 and 8 in MNHN.F.MAP327a (Figure 8). None of the studied specimens show a structure comparable to the “shiny strip of coalified material” reported by Schaumberg et al. (2007, p. 162) in association with the anterior patagials of the *Glaurung* holotype.

#### 3.3.2 External surface

**FIGURE 10:**
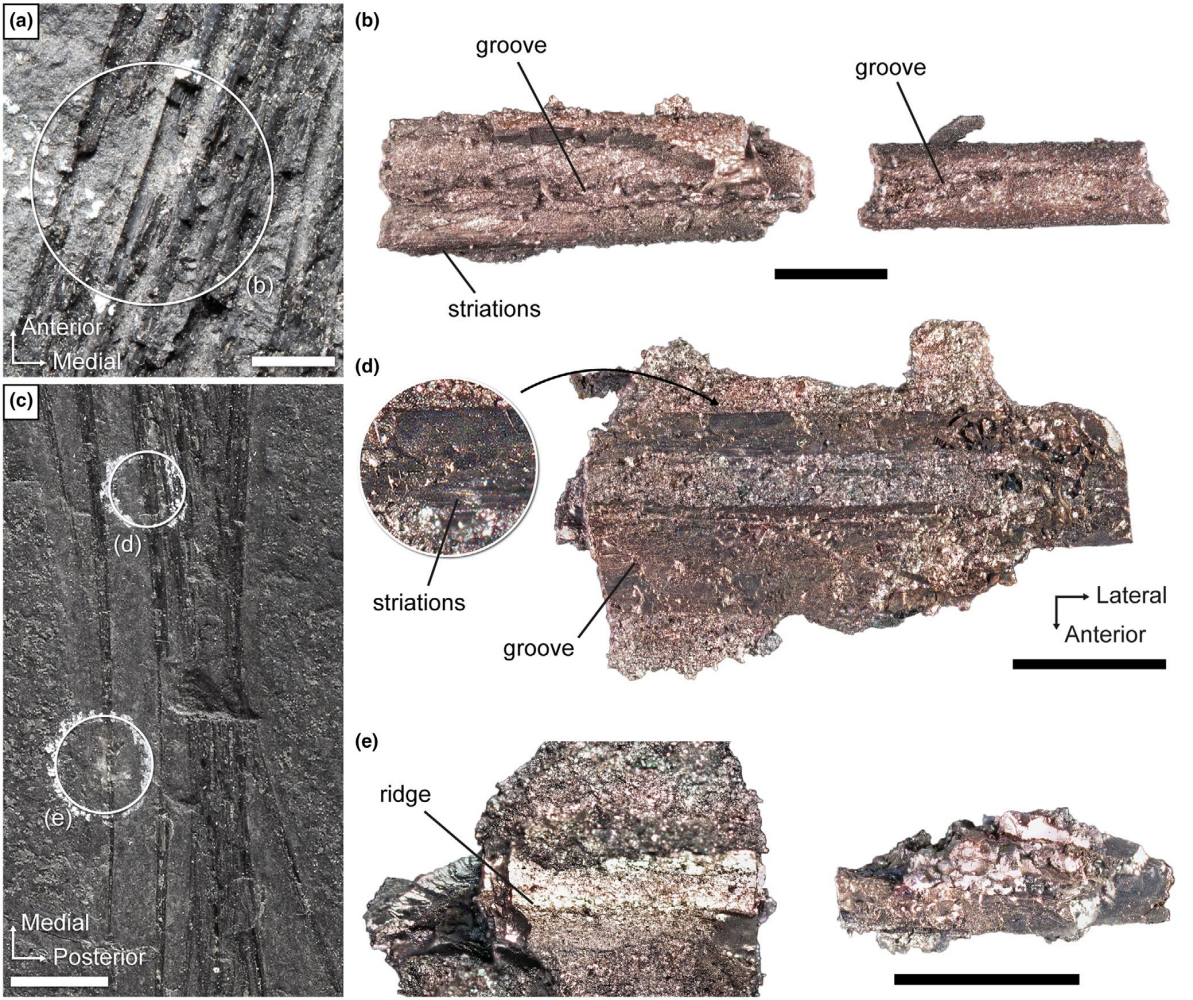
*Weigeltisaurus* sp. Kuhn, 1939 (Germany, Wuchiapingian). (a, b) Counterpart of Bodental specimen SMNK‐PAL 34866b. Close‐up (a) of the right wing showing the location of extracted mid‐length patagial fragments (b). (c–e) SMNK‐PAL 24865. Close‐up photograph (c) of the right wing showing the location of extracted mid‐length (d) and distal (e) patagial fragments. Scale bars: 10 mm (c), 2 mm (a), 1 mm (d, e), 500 μm (b).

We present here the first detailed examination of the external surface of the weigeltisaurids patagium based on the exquisitely preserved *Coelurosauravus* specimen MNHN.F.MAP327a (Figure 8) as well as fragments of patagials extracted from the *Weigeltisaurus* specimens SMNK‐PAL 34865 and SMNK‐PAL 34866b, the counterpart of the Bodental specimen (Figure 10).

Nearly all patagials of MNHN.F.MAP327a, excluding the most anterior and posterior ones, show a longitudinal groove running along the length of their proximal and middle thirds (Figure 8b,c,f). Such grooves are clearly seen in the mid‐patagial fragments extracted from the Bodental specimen SMNK‐PAL 34866b (Figure 10b), as well as in SMNK‐PAL 34865 based on the CT scan data of the proximal region of the patagials (Figure 3b,c), and more subtly based on the extracted mid‐patagial fragments (Figure 10d). The patagials of all weigeltisaurids thus have a pair of longitudinal contralateral grooves, giving them a biconcave cross section, at least in their proximal two‐thirds. However, the distal fragments extracted from SMNK‐PAL 34865 exhibit a low longitudinal ridge (Figure 10e), at least in the anterior patagials, whereas this region appears smooth in the anterior patagials of *Coelurosauravus* (Figure 8e). It is possible that this ridge in SMNK‐PAL 34865 results from subtle postmortem compression.

Our examination of the fragments extracted from SMNK‐PAL 24865 and SMNK‐PAL 34866b shows that, at least at mid‐length, the anterior and posterior margins of the patagials bore fine, longitudinal striations (Figure 10b,d). Such striations are also clearly visible at the base of the patagials of MNHN.F.MAP327a (Figure 8f). We suggest that these striations indicate the insertion for the patagial musculature and/or ligamentous connections between adjacent patagials (see Discussion below).

### 3.4 Histology of the abdominal skeleton of SMNK‐PAL 34865

#### 3.4.1 Anterior dorsal rib

**FIGURE 11:**
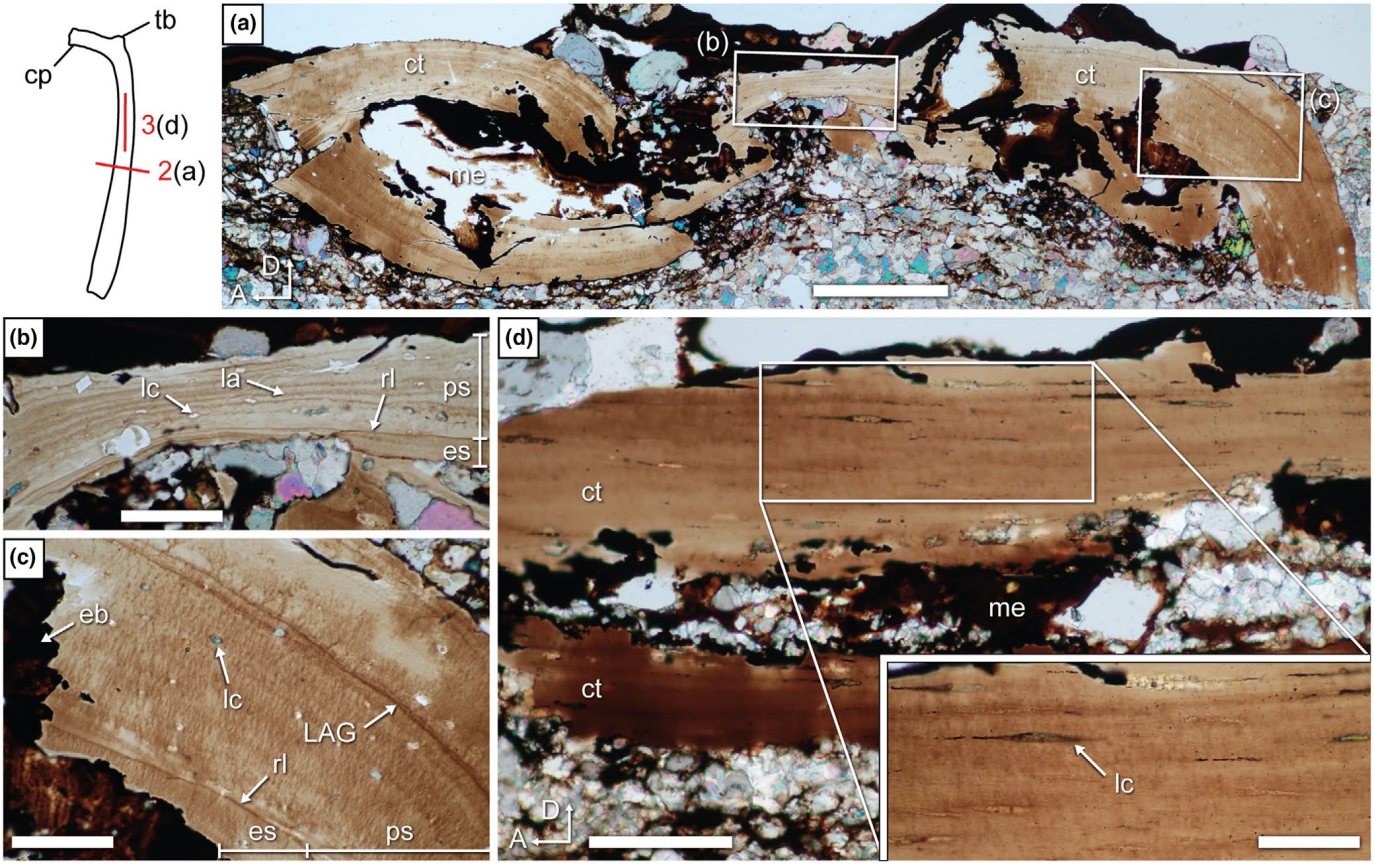
*Weigeltisaurus* sp. Kuhn, 1939 (Germany, Wuchiapingian) SMNK‐PAL 34865, thin sections 2 and 3 of anterior rib with line drawing illustrating location and direction of thin sections (red numbered lines). (a) Transverse section 2 through rib shaft at mid‐length with close‐ups showing lamellae (b), and annuli and osteocyte lacunae (c). (d) Longitudinal section 3 in proximal shaft. Inset, close‐up view of the spindle‐shaped osteocyte lacunae. cp, capitulum; ct, cortex; eb, erosion bay; es, endosteal bone; la, lamella; LAG, line of arrested growth; lc, osteocyte lacuna; me, medullary cavity; ps, periosteal bone; rl, reversion line; tb, tuberculum. Scale bars, 200 μm (a), 100 μm (d (main)), 50 μm (b, c, d (inset)).

The only endochondral bones sampled for thin sections so far in weigeltisaurids are the femur sectioned by Schaumberg et al. (2007) and the rib of SMNK‐PAL 34865 sectioned for this study (Figure 11). We produced a longitudinal section through the shaft of a rib of SMNK‐PAL 34865 and three transverse sections through the head and shaft of the same element (Sections 2–5; Figure 2b,c). Unfortunately, sections 4 and 5 through the rib heads and proximal shaft do not allow for histological description due to the poor preservation of the specimen.

As seen in transverse section 2, the rib shaft cortex is compact (Figure 11a). The dorsal and ventral portions of the cortex are relatively thin (ca. 67 μm) compared to the thicker cortex of the posterior portion (ca. 170 μm), although this may be accentuated by postmortem crushing or abrasion of the bone. Despite the crushing of the rib, the medullary cavity appears wide and lacks trabeculae. The cortex shows several randomly placed small ovate openings with a maximum diameter ranging between 3.5 and 6.5 μm (Figure 11c). These openings are much smaller than expected for vascular canals (20–50 μm; de Buffrénil et al., 2021). Instead, their diameter conforms to the maximum size of the fusiform osteocyte lacunae that are clearly seen in longitudinal section 3. The long axis of these lacunae is parallel to the lamellae (Figure 11d). Schaumberg et al. (2007) described similar lacunae in the femur of the Brandt specimen and mentioned the presence of canaliculi. However, we found no trace of canaliculi in the anterior dorsal rib of SMNK‐PAL 34865 (Figure 11d).

The lacunae are all invariably ovate in transverse section and fusiform in longitudinal section. This is indicative of parallel‐fibered bone (de Buffrénil et al., 2021). In contrast, the cortical bone of a weigeltisaurid femur was previously described as lamellar bone (Schaumberg et al., 2007). Several thin (ca. 2.5 μm) lamellae can be seen in the dorsal portion of the cortex (Figure 11b), which suggests that this section is composed of lamellar bone. However, in lamellar bone, the lacunae are oriented in the same plane as the lamellae, but the latter have different orientations. As a result, the lacunae would not show such an invariable orientation in lamellar bone (de Buffrénil et al., 2021). Here, we interpret the entire cortex of the rib of SMNK‐PAL 34865 as composed of parallel‐fibered bone and suggest the same for the femur of the Brandt specimen (Schaumberg et al., 2007: figure 9).

The cortex is formed mostly by primary periosteal bone, with a thin secondary endosteal deposit separated by a reversion line (Figure 11b,c). The deep cortex shows evidence of perimedullar resorption that created large erosion bays. We concur with Canoville et al. (2021) that a thin endosteal deposit is also visible in the femur on the latter specimen (Schaumberg et al., 2007: Figure 9). A single line of arrested growth (LAG) is present, and it can be traced throughout the circumference of the bone (Figure 11c).

#### 3.4.2 Gastralia

**FIGURE 12:**
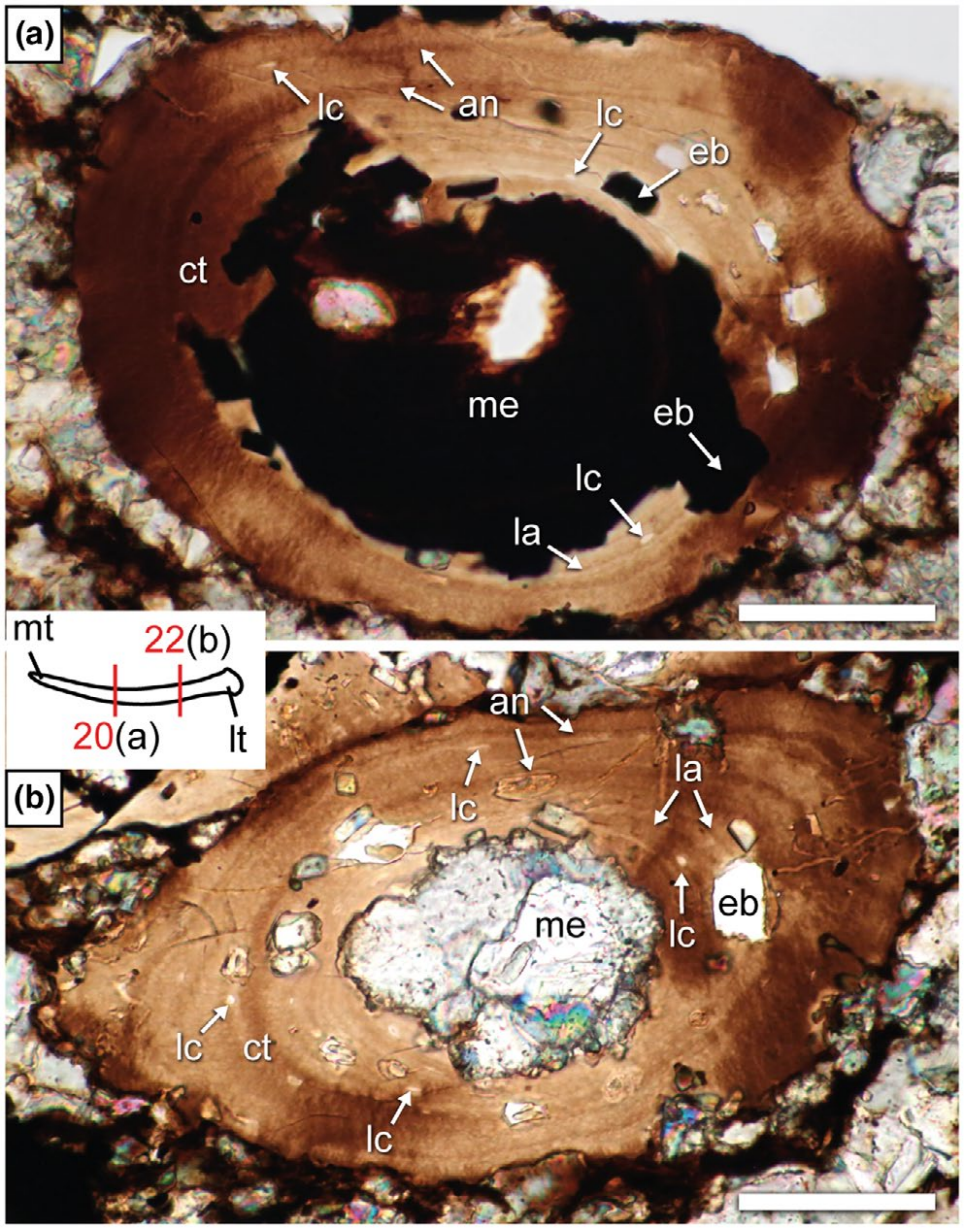
*Weigeltisaurus* sp. Kuhn, 1939 (Germany, Wuchiapingian) SMNK‐PAL 34865, fortuitously successively sampled gastralium embedded in matrix. Transverse thin sections 20 through medial (a) and 22 through lateral (b) portions of a single displaced gastralium. an, annulus‐like structure; ct, cortex; eb, erosion bay; la, lamella; lc, osteocyte lacuna; lt, lateral terminus; me, medullary cavity; mt, medial terminus. Scale bars, 50 μm.

The only unequivocal dermal bones sectioned so far in weigeltisaurids are the gastralia investigated here (Figure 12). These elements are very small and fragile, and most are very badly preserved in SMNK‐PAL 34865. As such, although some exposed gastralia were targeted (sections 6 and 8; Figure 2), their surface is badly preserved, which precludes microanatomical or histological description. Instead, we focus here on an underlying gastralium that is encased in the matrix below the patagials and is much better preserved (Figure 12). However, as these sections result from fortuitous sampling when targeting the patagials, they are not necessarily perfectly transverse. Nevertheless, these sections yield new information about the microanatomy and histology of the gastralia in weigeltisaurids.

**FIGURE 13:**
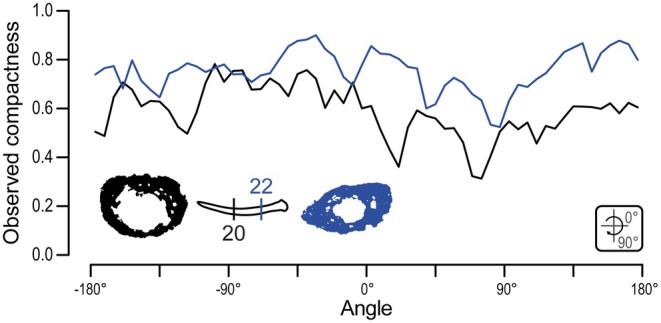
*Weigeltisaurus* sp. Kuhn, 1939 (Germany, Wuchiapingian) SMNK‐PAL 34865, radial profile of observed compactness for successive transverse sections of a single gastralium. Cortical thickness varies substantially around the sampled sections.

A single gastralium was successively sectioned in transverse sections 20 and 22 (Figure 12). This gastralium is ca. 215 μm in maximal diameter in section 20, with a medullary cavity of ca. 123 μm, and is ca. 230 μm wide with a ca. 81 μm wide medullary cavity in section 22. In line with this slight increase in maximum width, the gastralium is circular in cross section in section 20 but oval in section 22. As a result, the observed compactness is markedly higher in section 22 (between 0.52 and 0.90) than in section 20 (0.31–0.78) (Table 3; Figure 13). Given that gastralia gradually thicken laterally in all weigeltisaurids (Figures 3, 4, 5 and 8), we here consider that sections 20 and 22 represent successive sections of the same gastralium along its mediolateral length (Figures 12 and 13). Provided that this interpretation is correct, the medullary cavity appears to decrease in relative size from medial to lateral along the length of the gastralia in weigeltisaurids.

**TABLE 3:** Parameters of the compactness profile recovered by the BoneProfileR radial analyses of complete gastralia transverse sections and partial patagial transverse sections. Note that a single thin section can cut through several elements, which may correspond to different patagial portions (Figure 2b).

Section	Number of analyzed subsections	Minimum observed compactness	Maximum observed compactness
**Gastralia**			
Section 20	—	0.31	0.78
Section 22	—	0.52	0.90
**Medial part of patagial**			
Section 12	3	0.12	0.82
**Middle part of patagial**			
Section 17	4	0.27	0.84
Section 22	4	0.08	0.87
**Lateral part of patagial**			
Section 19	3	0.51	0.81
Section 22	3	0.39	0.90
**Lateral extremity of patagial**			
Section 17	4	0.69	0.89
Section 20	5	0.31	0.85
Section 22	5	0.17	0.81
Section 26	3	0.76	0.89

The gastralia of SMNK‐PAL 34865 are composed of a thick cortex of avascular bone marked by deep erosion bays and framing a medullary cavity devoid of trabeculae (Figure 12). Few circular or ellipsoid osteocyte lacunae are present and arranged along the lamellae. Similar to the anterior rib described above, no birefringence was found in the thin sections of the gastralia, suggesting that the cortex is also composed of parallel‐fibered bone. There is no trace of a secondary endosteal deposit (Figure 12). Two well‐marked annulus‐like structures are seen throughout the circumference of the bone on both sections 20 and 22.

#### 3.4.3 Patagials

Schaumberg et al. (2007) produced one transverse and one longitudinal section of patagials from the Brandt specimen. However, it is unclear if those sections correspond to anterior or posterior patagials (or both because they could come from distinct bones), and from which portion of a patagial (proximal, middle, or distal), these sections were made. Here, we provide an extensive histological sample from the anterior patagials preserved in SMNK‐PAL 34865 (Figure 2b) in order to describe histological variations along the length of a patagial bone.

We targeted the proximal extremity of several patagials in SMNK‐PAL 34865 (Sections 9–13). Unfortunately, these sections only offer limited histological information because this part of the specimen is poorly preserved. We then sampled the proximal (sections 1, 7, 14–16), middle (sections 17–19), and distal (sections 20–22) portions of the shafts, as well as distal extremities (sections 23–26). In all samples, at least one section is sufficiently well preserved for histological description.

**FIGURE 14:**
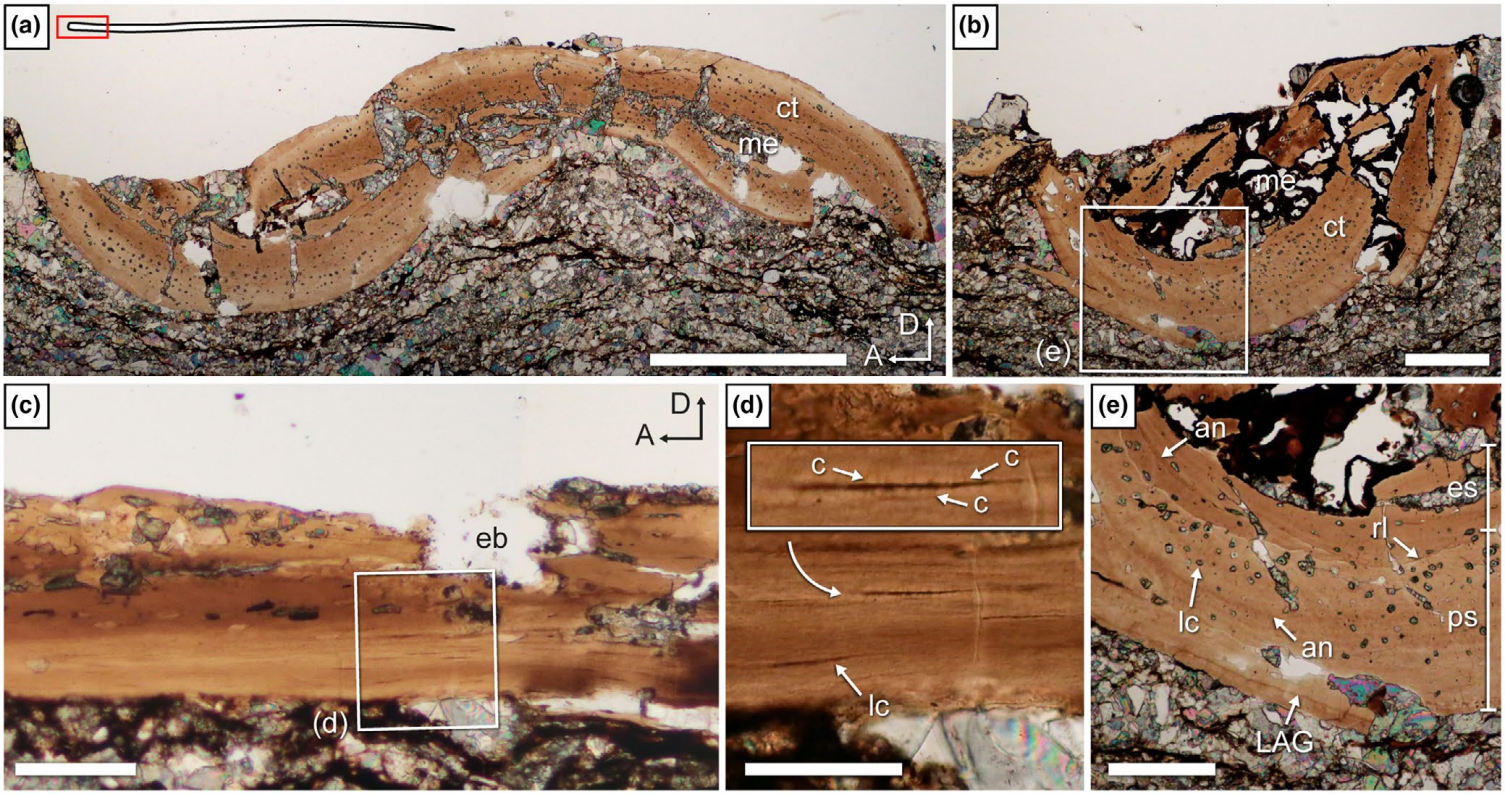
*Weigeltisaurus* sp. Kuhn, 1939 (Germany, Wuchiapingian) SMNK‐PAL 34865, thin sections 13 (a, b, e) and 11 (c, d) through the proximal extremity of patagials. (a, b) Portions of the transverse section 13. (c) Longitudinal section 11 with a close‐up (d) showing fusiform osteocyte lacunae with canaliculi. (e) Close‐up of (b). an, annulus‐like structure; c, canaliculi; ct, cortex; eb, erosion bay; es, endosteal bone; LAG, line of arrested growth; lc, osteocyte lacuna; me, medullary cavity; ps, periosteal bone; rl, reversion line. Scale bars 500 μm (a), 200 μm (b), 100 μm (c, e), 50 μm (d).

Several proximal extremities of patagials were sectioned in transverse section 13, showing that the cortex is composed of thick avascular parallel‐fibered bone, with a free medullary cavity showing evidence of perimedullar resorption and erosion bays (Figure 14). Unfortunately, all of the proximal extremities of the patagials are crushed, precluding an assessment of the cortical thickness and size of the medullary cavity. The cortex is divided into primary periosteal bone and a thinner secondary endosteal deposit separated by a scalloped reversion line (Figure 14e). A single well‐marked LAG is seen in the outer cortex, and the periosteal and endosteal regions each show a single annulus‐like structure. Additionally, transverse section 13 indicates the presence of lacunae with greatest diameters of 4.5–8 μm (Figure 14e), similar to the condition in the dorsal rib. At first glance, this does not seem to conform to the extremely thin (ca. 2 μm) fusiform lacunae which are visible in longitudinal section 11, but we think that this is due to the postmortem compression visible on the latter section (Figure 14c,d). Nevertheless, the osteocyte lacunae show very short (1.5–1.8 μm long) canaliculi running perpendicular to the main axis of the lacuna (Figure 14d). This is similar to the canaliculi described by Schaumberg et al. (2007) for the Brandt specimen.

**FIGURE 15:**
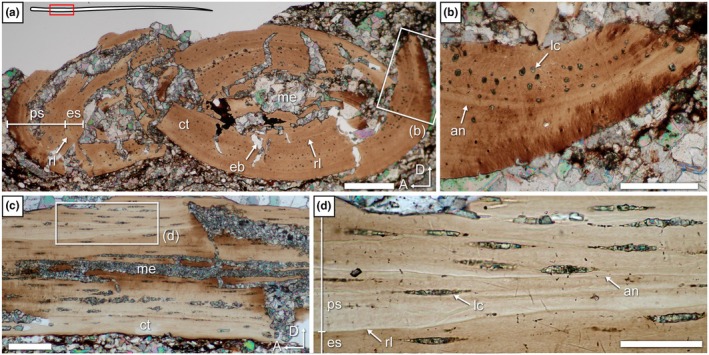
*Weigeltisaurus* sp. Kuhn, 1939 (Germany, Wuchiapingian) SMNK‐PAL 34865, thin sections 15 (a, b) and 7 (c, d) through the proximal part of the shaft of patagials. (a) Transverse section 15 with close‐up (b) showing osteocyte lacunae. (c) Longitudinal section 7 with close‐up showing fusiform osteocyte lacunae. an, annulus‐like structure; ct, cortex; es, endosteal bone; lc, osteocyte lacuna; me, medullary cavity; ps, periosteal bone; rl, reversion line. Scale bars 200 μm (a), 100 μm (b, c), 50 μm (d).

**FIGURE 16:**
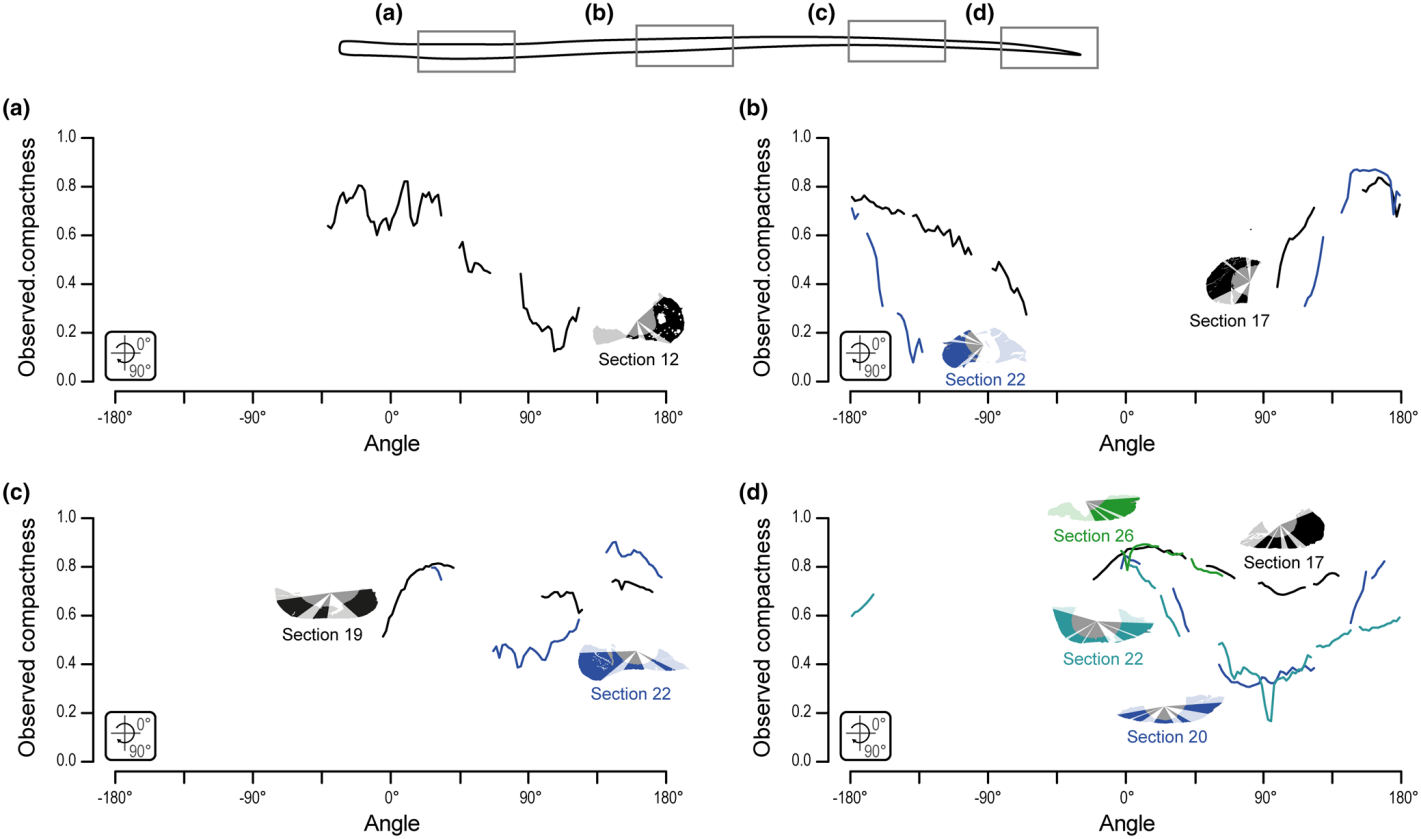
*Weigeltisaurus* sp. Kuhn, 1939 (Germany, Wuchiapingian) SMNK‐PAL 34865, radial profiles of observed compactness for the transverse section through the medial (a), middle (b), and lateral (c) portions of patagial shafts and through the lateral extremity of patagials (d). Note that a single thin section can cut through several elements, which may correspond to different patagial portions (Figure 2b). The sampled sections clearly show variation in cortical thickness: Observed compactness is lower around 90 and −90° (along the short axis of the section) and higher around 0 and 180° (along the long axis).

As seen in sections 15 and 7 (transverse and longitudinal sections, respectively), the proximal parts of the patagial shafts are composed of a thick cortex of avascular bone, with a medullary cavity showing evidence of perimedullar resorption and erosion bays (Figure 15). A thin secondary endosteal deposit is separated from primary periosteal bone by a reversion line, similar to the condition in the proximal extremity of the patagials (Figure 15c,d). Compared to the dorsal rib, the proximal patagial shafts of SMNK‐PAL 34865 have a much thicker cortex, so the medullary cavity appears to be much smaller (Figure 15a,c). Yet, the thickness of the cortex itself varies, being thick (observed compactness ca. 0.80) along the long axis of the cross section but thin (observed compactness 0.12) along the short axis (Table 3; Figure 16a). Annulus‐like structures are seen in the anterior and posterior portions of the bone in transverse section 12 (Figure 15b). There is no trace of LAGs, in contrast to the proximal extremity of the patagials. As seen in longitudinal section 7 (Figure 15d), the proximal parts of the patagial shafts of SMNK‐PAL 34865 contain several thin (3.5–5.5 μm tall) fusiform osteocyte lacunae arranged conformably along the lamellae. We could not identify any canaliculi, which are present in the proximal extremity of the patagials, but this could be due to the preservation of the bone because all lacunae observed in the proximal portion of the shafts appear to be filled with mineral.

**FIGURE 17:**
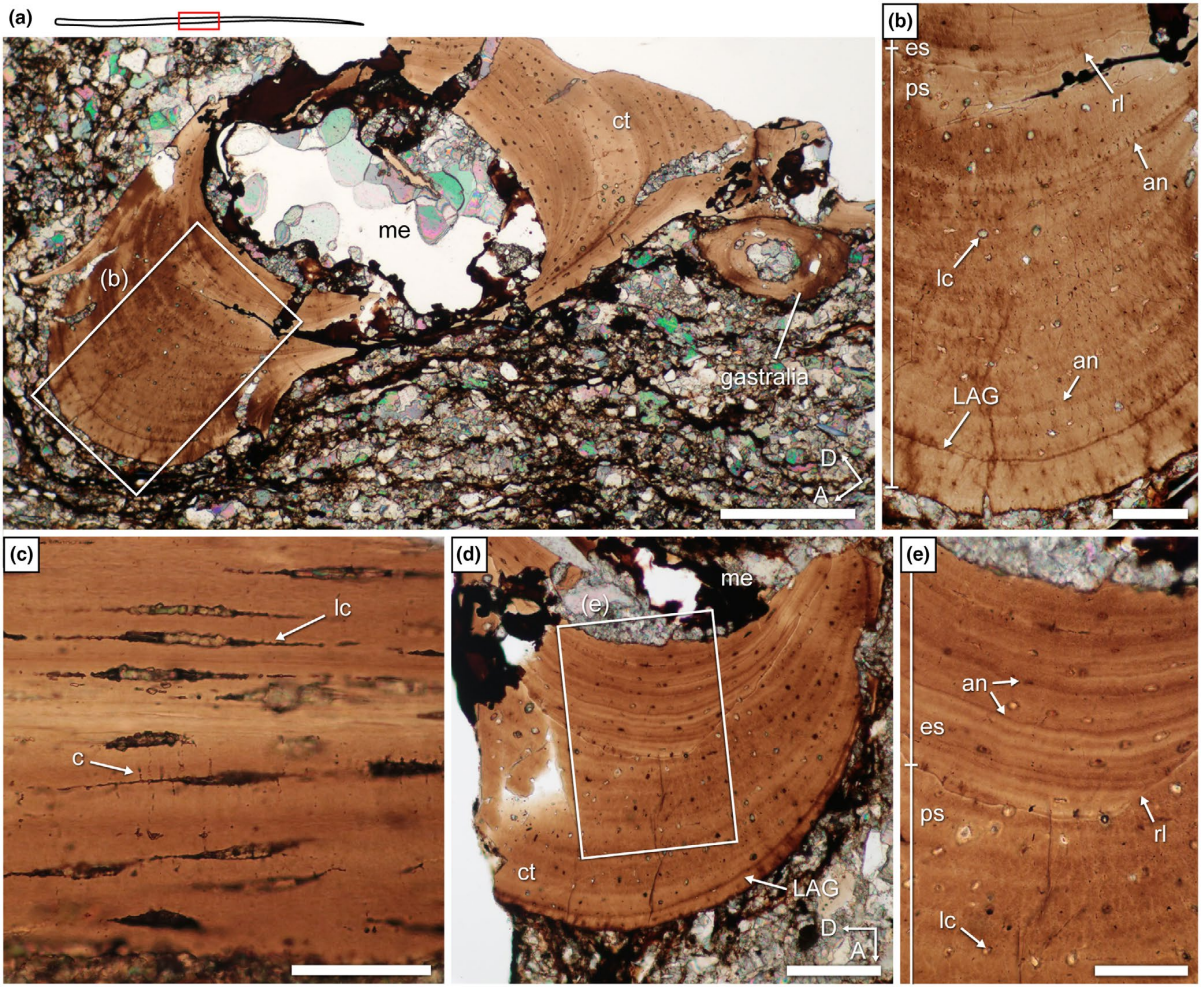
*Weigeltisaurus* sp. Kuhn, 1939 (Germany, Wuchiapingian) SMNK‐PAL 34865, thin sections 22 (a, b), 18 (c), and 19 (d, e) through middle portion of shaft of patagials. (a) Transverse section 22 with close‐up (b). (c) Close‐up of longitudinal section 18 showing fusiform osteocyte lacunae with canaliculi. (d) Transverse section 19 with close‐up (e) showing resorption line and centripetal endosteal deposit. an, annulus‐like structure; c, canaliculi; ct, cortex; es, endosteal bone; LAG, line of arrested growth; lc, osteocyte lacuna; me, medullary cavity; ps, periosteal bone; rl, reversion line. Scale bars, 200 μm (a), 100 μm (d), 50 μm (b, c, e).

The middle portion of the patagials is the best‐preserved part of these bones in SMNK‐PAL 34865. Transverse section 22 (Figure 17a) shows a subcomplete patagial that clearly illustrates the ovoid and biconcave cross‐sectional shape of the patagials that was previously reconstructed by Schaumberg et al. (2007). As reconstructed, we estimate that the bone is more than four times as wide (ca. 1100 μm along the main axis of the section) as tall (ca. 255 μm). The medullary cavity is also ovate but is instead slightly taller (ca. 455 μm) than wide (ca. 410 μm). In line with this, the observed compactness is very high along the long axis of the section (ca. 0.85) but drops rapidly toward its short axis (<0.1) (Table 3; Figure 16b). The cross‐sectional shape of the bone thus results from an extreme difference in cortical thickness, with the anterior and posterior walls being approximately eight times thicker than the dorsal and ventral ones and with the medullary cavity occupying about a third of the transverse diameter of the bone (Figure 17a). As seen in transverse sections 19 and 22, the cortex comprises a thick secondary endosteal deposit separated by a resorption line from the 1.5 to 4 times thicker primary periosteal bone (Figure 17b,e). Several concentric annulus‐like structures are seen in both the periosteal and endosteal bone, and one LAG is visible in the outer cortex. Lastly, as seen in longitudinal section 18 (Figure 17c), some short (5–7 μm long) canaliculi extend perpendicular to the long axis of the fusiform lacunae. Similar canaliculi were described by Schaumberg et al. (2007) for the Brandt specimen, and their preservation in both the proximal extremity and the middle portions of the patagials in SMNK‐PAL 34865 suggests that they were likely present in the proximal patagial shafts too, and possibly also in the dorsal rib sectioned here, but are obscured by mineralization.

**FIGURE 18:**
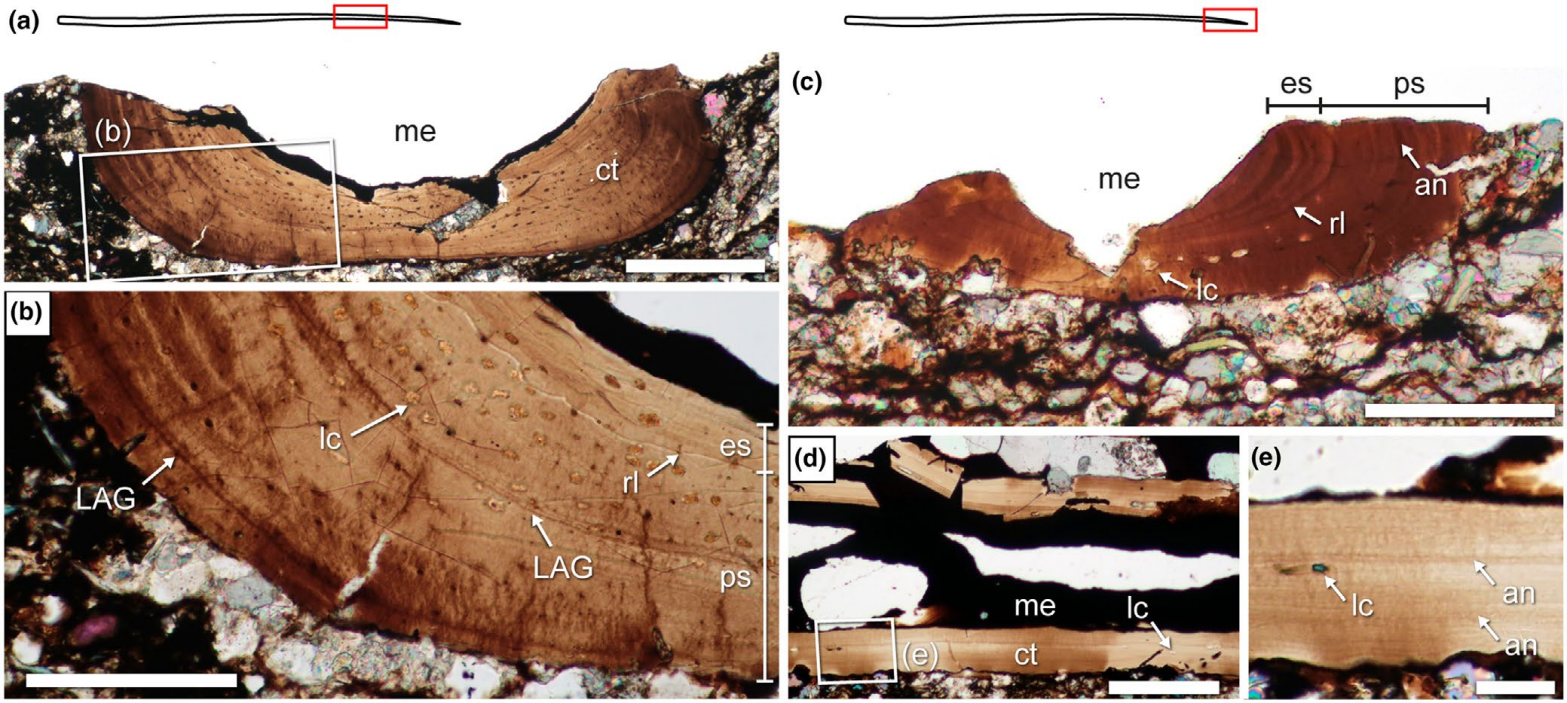
*Weigeltisaurus* sp. Kuhn, 1939 (Germany, Wuchiapingian) SMNK‐PAL 34865, thin sections 19 (a, b) through distal portion of shaft of a patagial, and 26 (c) and 23 (d, e) in distal extremity of a patagial. (a) Transverse section 19 with close‐up (b). (c) Transverse section 26. (d) Longitudinal section 23 with close‐up (e). an, annulus‐like structure; ct, cortex; es, endosteal bone; LAG, line of arrested growth; lc, osteocyte lacuna; me, medullary cavity; ps, periosteal bone; rl, reversion line. Scale bars, 200 μm (a), 100 μm (c, d), 50 μm (b), 20 μm (e).

Transverse section 19 shows the distal portion of a patagial shaft (Figure 18a). This section is only subtly oval compared to the middle portion of the patagial shafts. We estimate it to be about 1.8 times as wide (ca. 905 μm along its long axis) as tall (ca. 500 μm), with a similarly shaped ca. 530 μm wide medullary cavity that occupies more than half of the transverse diameter of the bone. Consistent with this shape, the periosteal and endosteal portions are much thinner than in the middle of the patagials (Figure 18b). In line with this, the observed compactness remains higher (ca. 0.4) along the short axis of the section compared to the middle portion of the patagial shafts (Table 3; Figure 16c). The cortex is composed of avascular parallel‐fibered bone with a medullary cavity devoid of trabeculae, as in the proximal and middle portions of the patagial shafts. Several osteocyte lacunae are seen in the deeper half of the periosteal cortex and, with a lesser density, in the outer periosteal and endosteal regions. This contrasts with the more proximal sections of the patagials where the lacunae appear to be randomly distributed (Figures 14, 15 and 17). Two strongly marked LAGs are seen in the periosteal cortex, one in the outer cortex, and one marking the onset of the lamellae where the lacunae are concentrated (Figure 18b). None of the longitudinal sections provide any significant histological information about the distal portion of the patagials.

A partial distal extremity of a patagial is seen in transverse section 26 (Figure 18c). We tentatively estimate this bone to be 380 μm wide and 190 μm tall. The section is thus subtly oval in outline, as in the distal portion of the shaft, but with a smaller medullary cavity. The observed compactness remains very high (>0.80) along the long axis of the section but decreases along the short axis of the bone to a sharper degree (down to ca. 0.2) than in the lateral part of the shaft (Table 3; Figure 16d). We identify a resorption line in the deep third of the cortex based on its slightly irregular shape, especially where the cortex is thickest, and the much more distinct annulus‐like structures deep to it, similar to the condition in the mid‐patagial cross section 19 (Figure 17d). The bone cortex thus still comprises distinct endosteal and periosteal deposits, both of which show annulus‐like structures. In transverse section 26 (Figure 18c), few lacunae are seen and appear to be aligned along a single layer in the deeper periosteal cortex. Longitudinal section 23 shows the distal tip of a patagial (Figure 18d). The cortex is extremely thin (ca. 91 μm), so the medullary cavity (ca. 57 μm) occupies more than half of the bone diameter. This longitudinal section shows a few fusiform osteocyte lacunae (Figure 18d,e), indicating that the cortex of the distal extremity of the patagials remains composed of parallel‐fibered bone. Two well‐marked annulus‐like structures are visible in longitudinal section 23 (Figure 18d,e).

## 4 DISCUSSION

### 4.1 Homology of the patagials

As detailed above, the homology of the patagial skeleton of weigeltisaurids has been extensively discussed since the recognition of their gliding ability. The current consensus is that the patagials are dermal ossifications and not derivatives of the endochondral skeleton (Buffa et al., 2022; Frey et al., 1997; Pritchard et al., 2021; Schaumberg, 1976, 1986; Schaumberg et al., 2007). However, their exact homology remains unclear. Above, we provided evidence for a one‐to‐one articulation between patagials and lateral gastralia. Thus, we confirm Pritchard et al.'s (2021) and Buffa et al.'s (2022) interpretations that the patagial skeleton is linked to the rest of the body at the ventrolateral margin of the trunk, level with and articulated to the gastral basket.

Based on this interpretation, Pritchard et al. (2021) recently identified three possible interpretations for the homology of the patagials: (i) intermuscular ossifications that formed within the myosepta of external trunk musculature; (ii) modified lateral gastralia; (iii) true neomorphs without homology with any pre‐existing soft or hard tissues. Of these hypotheses, Pritchard et al. (2021) tentatively supported an identification of patagials as true neomorphs, whereas Buffa et al. (2022) tentatively considered them as lateral gastralia.

Neither Pritchard et al. (2021) nor Buffa et al. (2022) supported the interpretation that the patagials are intermuscular ossifications that formed within the myosepta of external trunk musculature. Such ossifications are present in a variety of teleosteans (Danos & Ward, 2012; Gemballa & Britz, 1998; Patterson & Johnson, 1995) and among tetrapods only in the tail of pachycephalosaurian dinosaurs (Brown & Russell, 2012). However, contrary to the tail, the trunk myosepta of tetrapods coalesce during development, obscuring the plesiomorphic segmental architecture seen in teleosts (Gemballa & Ebmeyer, 2003). As a result, it is extremely unlikely that intermuscular bones corresponding to the ancestral myoseptal segmentation of the trunk would be present in weigeltisaurids.

Buffa et al. (2022) tentatively suggested that patagials might be modified lateral gastralia due to their close association with unequivocal gastralia that resemble the articulation between successive elements in gastral rows in other amniotes (e.g., Buffa et al., 2025). *Coelurosauravus* has two pairs of unequivocal gastralia per row, the maximum number in any weigeltisaurid. This is one less than the three parasagittal pairs of elements that are typically found in millerettids, non‐saurian neodiapsids, and early saurians (Buffa et al., 2025; Jenkins et al., 2025). Based on external morphology alone, it would be parsimonious to consider patagials as the most lateral pairs of each gastral row because this would still be consistent with the typical number of gastralia in coeval reptiles.

However, in extant reptiles, the end of each dorsal rib is connected to the lateral tip of at least one of the lateral gastralia through connective tissue in the body wall (Daiber, 1920; Howes & Swinnerton, 1901). This was probably also the case in stem‐saurian reptiles, including weigeltisaurids, which is a strong argument against the homology between gastralia and patagials. Given the large wingspan of weigeltisaurids, such a connection between the lateral tip of the patagials and the dorsal ribs is extremely unlikely. This connection would also restrict the expansion of the wing and thus gliding performance. Instead, we suggest that the dorsal ribs were likely connected to the expanded lateral heads of the unambiguous lateral gastralia, with connective tissues possibly inserting on either or both of the dorsal tubers described above (Figure 5c,d). Under this interpretation, it is unlikely that the patagials and gastralia are strictly homologous.

**FIGURE 19:**
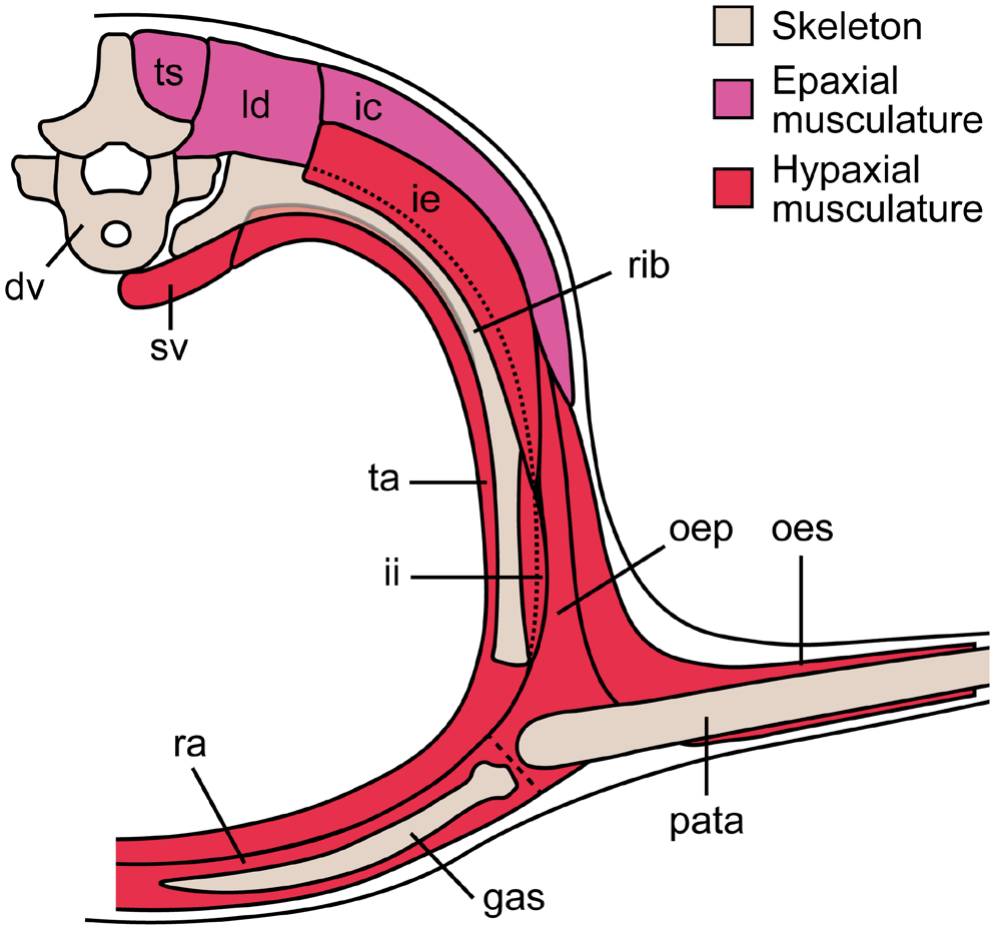
Reconstruction of the weigeltisaurid trunk musculoskeleton in cross section. dv, dorsal vertebra; ga, gastralia; ic, M. iliocostalis; ie, M. intercostralis externus; ii, M. intercostalis intenus; ld, M. longissimus dorsi; ope, M. obliquus externus profundus; oes, M. obliquus externus superficialis; pata, patagial; ra, M. rectus abdominis; sv, M. subvertebralis; ta, M. transversus abdominis; ts, M. transversospinalis.

The musculoskeletal relationships of the patagial skeleton have some bearing on the assessment of their homology. In *Sphenodon* and crocodilians, the only extant saurians with unambiguous gastralia, these bones are mostly embedded in the superficial layers of the M. rectus abdominis, eventually with smaller insertions of the M. obliquus externus group at least on the lateral elements of the most posterior rows (Byerly, 1925; Claessens, 2004; Vickaryous & Hall, 2008). Due to their location, gastralia play a role in protecting the viscera (Romer, 1956), in preventing abdominal collapse (Perry, 1983), and in ventilation and breathing (Claessens, 2004, 2009) in extant reptiles, and presumably in extinct ones too. Therefore, it is likely that the unambiguous gastralia of weigeltisaurids, located in the trunk, were also embedded in the M. rectus abdominis (Figure 19) and fulfilled similar functions. Conversely, patagials, which can extend up to 166 mm lateral to the abdominal wall, seem ill‐suited for such biological functions. It is thus extremely unlikely that these bones were embedded in the M. rectus abdominis throughout their length. Rather, we suggest that they served as the insertion for fibers of the M. obliquus externus group, the most external layers of the abdominal wall musculature, with its superficial part inserting some distance along the length of the patagials (Figure 19). The M. obliquus externus group, in particular its superficial part, connects the skeletal elements and skin, and is typically involved more in trunk movements during locomotion than in ventilation in extant reptiles (Gasc, 1981). As a matter of fact, the M. obliquus externus is also highly modified in *Draco*, in line with the acquisition of a patagium supported by the dorsal ribs (John, 1970), indicating that this muscle can be mobilized in gliding locomotion without jeopardizing ventilation capacity in reptiles.

However, we note that the gastralia of extant reptiles initially develop external to the M. rectus abdominis before becoming embedded in its superficial layer later on during prenatal development (Howes & Swinnerton, 1901; Vickaryous & Hall, 2008). Supposing that patagials are modified lateral gastralia, it could be argued that only the medial gastral pairs got embedded in the M. rectus abdominis, or even that the degree of embedding of the patagials in this muscle was drastically reduced.

The marked histological differences described above between gastralia and patagials provide another strong argument against a strict homology of these bones in weigeltisaurids. The patagials are composed of a thick cortex of parallel‐fibered bone showing evidence of perimedullar resorption and endosteal deposition (Figures 14, 15, 16, 17, 18), which is similar to the condition in endochondral bones such as dorsal ribs and femora, but contrasts with the gastralia which lack endosteal deposits (Figure 12). In addition, the patagials show a highly asymmetrical cortex that is not found in any other bone (Figure 16), including the gastralia, which show much more homogeneously compact cortices (Figure 13). This further suggests that patagials are not modified lateral gastralia. It could be argued that this is simply due to the size differences between the two bone types because patagials are generally at least one order of magnitude larger than gastralia. However, the very thin distal extremity of the patagials, which is closer in size to the gastralia, also appears to retain an endosteal deposit as well as a marked asymmetry in compactness (Figures 16d and 18c–e). Thus, we positively reject the hypothesis that gastralia and patagials are strictly homologous.

In light of all this, we are inclined to support the idea that patagials are true neomorphs, as previously suggested by Frey et al. (1997) and Pritchard et al. (2021). We could not find any analog bones for the weigeltisaurid patagials in both extinct and extant tetrapods, and we note that the presence of a medullary cavity and primary periosteal bone differs from the general morphology described for osteoderms (Pochat‐Cottilloux et al., 2023; Scheyer et al., 2014) or calcified tendons (Klein et al., 2012; Organ & Adams, 2005) in other extinct reptiles. This suggests that the patagials are not derived from pre‐existing unossified tendons, in agreement with Canoville et al. (2021).

As a result, weigeltisaurids show a reduction in the number of gastralia per gastral row from the plesiomorphic state of three elements per side in neodiapsids (Buffa et al., 2025; Jenkins et al., 2025) to two in *Coelurosauravus* (Figure 8) and one in *Weigeltisaurus* (Figures 2, 3, 4, 5, 6, 7). Among terrestrial Permo‐Triassic reptiles, similar reductions are also found in the millerettid *Milleretta* (Gow, 1997), drepanosauromorphs (Buffa, Frey, et al., 2024a; Renesto et al., 2010; Spiekman et al., 2025), testudines (possibly including the enigmatic *Eunotosaurus*; Lyson et al., 2013; Schoch & Sues, 2020), squamates (Tałanda et al., 2022), and archosaurs (Claessens, 2004; Fechner & Gößling, 2014; Radermacher et al., 2021).

Lastly, our identification of patagials as neomorphs raises the question of their morphogenetic origin and development. Given their location immediately lateral to the body wall and articulation to the gastralia, the patagials may also develop initially inside the loose mesenchyme that frames the body wall. In particular, the topographical position of the patagials suggests that they form in the region of the lateral fold, which is incidentally extensively developed in some extant gliding geckos, forming their main patagium (Russell et al., 2001). This would place the weigeltisaurid patagials in the expected position to receive the insertions of the M. obliquus externus group, as is the case for the most posterolateral gastralia in modern reptiles (Byerly, 1925; Claessens, 2004; Vickaryous & Hall, 2008). Under this hypothesis, patagials would still be neomorphic bones, but their osteogenesis would rely on a conserved morphogenetic system. Thus, they may ossify in a posterior to anterior sequence, as occurs for the gastralia (Howes & Swinnerton, 1901; Vickaryous & Hall, 2008). This hypothesis cannot be tested at present as even the smallest (and thus presumably ontogenetically youngest) weigeltisaurid specimen, the Wolfsberg specimen, already shows completely ossified gastralia and patagials (Figure 7c–e).

### 4.2 Musculoskeletal relationships of the patagials and implications for wing unfolding mechanism

As in our previous work (Buffa et al., 2022), we are skeptical of Schaumberg et al.'s (2007) hypothesis of a tendinous connection between the scapula and anterior‐most patagial, whereby the wing could have been extended by a sharp protraction of the forelimb and could be further stabilized by filling the lungs with air to inflate the ribcage. We found no evidence for such a tendinous articulation, which would have covered a long distance over the anterior trunk due to the presence of a cartilaginous sternum (not reconstructed by Schaumberg et al., 2007). Furthermore, extension of the patagia through protraction of the forelimb appears unlikely because it could cause accidental openings of the patagia during limb‐based locomotion. Lastly, the available material does not warrant discussing the benefits of inflating the lungs and holding the breadth during gliding.

The new anatomical data and homology statement on the patagials detailed above allow for the reassessment of the wing unfolding mechanism in weigeltisaurids based on the reconstruction of their trunk and wing skeleton and musculature (Figure 19) and comparisons to the flying lizard *Draco*, the best extant analog for weigeltisaurids, whose wing anatomy and unfolding mechanism are well known.

In the flying lizard *Draco*, the abdominal musculature is highly specialized in line with gliding locomotion (Colbert, 1967; John, 1970; Russell & Dijkstra, 2001). Of the epaxial musculature, the M. iliocostalis is stronger anteriorly compared to non‐gliding squamates, inserting over large areas on the anterior surface of the first two patagial ribs. This reinforced part of the M. iliocostalis serves to unfold the patagium by pulling on these anterior ribs, which are linked to the following ones by intercostal muscles and ligaments (Colbert, 1967; Russell & Dijkstra, 2001). Because the patagium of weigeltisaurids is supported by the patagials, which are not homologous to the dorsal ribs and are located much more ventral than them, the iliocostal and intercostal muscles topographically could not have been involved in the operating of the patagium, contrary to the condition in *Draco*. Because these muscles indeed invariably attach to the dorsal ribs in extant reptiles (Gasc, 1981), there is no reason to think that this was not the case in weigeltisaurids. Instead, as discussed above, the patagials probably served as insertions for the M. obliquus externus group (Figure 19).

In *Draco*, the M. obliquus externus is very reduced compared to the condition in non‐gliding squamates and does not form the outermost layer of the body wall. Instead, it forms a “rim muscle” (John, 1970, p. 161) lining the rim of the patagium and linking the lateral tips of the patagial ribs to the sternum. Thus, the contraction of the M. obliquus externus contributes to the unfolding of the patagium. The intercostal musculature is also highly reduced and specialized in the patagial region, connecting the individual patagial ribs instead of contributing to the abdominal wall as in non‐gliding squamates (Colbert, 1967; John, 1970; Russell & Dijkstra, 2001). The more medial M. intercostalis externus serves to unfold the patagium, whereas the more lateral M. intercostalis internus contributes to folding it. Overall, the hypaxial musculature is very reduced in mass compared to non‐gliding squamates, including the M. rectus abdominis. This contributes to a reduction of body weight while still permitting efficient operation of the patagium (John, 1970).

By analogy with *Draco*, the M. obliquus externus group likely also contributed to the unfolding of the patagium in weigeltisaurids through its insertions on the patagials. Its deep part may have inserted near the base of the patagials, while the superficial part could have inserted at least some distance over the length of the patagials (Figure 19). These insertions could be evidenced by the striations seen over the external surface of the patagial spars of *Weigeltisaurus* (Figure 10). However, these striations could also suggest the presence of interpatagial ligaments. Such ligaments would have mechanically made the patagium a functional unit, analogous to the condition in *Draco* (Colbert, 1967; Russell & Dijkstra, 2001). It remains unclear, however, if the M. obliquus externus group, namely its superficial part, framed the perimeter of the patagium as it does in *Draco*. Contrary to *Draco*, it is unlikely that the M. intercostalis externus contributed to the wing unfolding mechanism, given that the dorsal ribs are not involved in the weigeltisaurid patagium.

In *Draco*, the patagial musculature alone does not allow for the complete unfolding of the patagium, which is primarily unfolded by pulling on the leading edge using the forelimbs and manual claws (Dehling, 2017). As previously proposed (Buffa et al., 2022), the unfolding of the wing in weigeltisaurids was also most likely conducted by interlocking the manual claws into the scales of the leading edge of the wing. This would require postaxial abduction of the manus, which appears to have been possible in weigeltisaurids based on the position of the articulated right manus of the Ellrich specimen (Figure 1). The forelimbs of weigeltisaurids could extend over half of the length of the leading edge of the wings, further facilitated by the presence of an additional phalanx in digit V (Buffa et al., 2022; Bulanov & Sennikov, 2010; Pritchard et al., 2021).

Regarding the folding mechanism, we did not find any evidence of muscle insertions indicating the role of a specific muscle in folding, as is the case for the M. intercostalis internus in *Draco* (John, 1970). It is possible that the patagium in weigeltisaurids was folded solely by collagen‐ and elastin‐rich connective tissue that passively restricted the expansion of the wing by linking the distal tips of the patagials along the margin of the wing, as is the case in *Draco* (Russell & Dijkstra, 2001), but this cannot be demonstrated based on the currently available material.

### 4.3 Biomechanical properties of the patagial skeleton

#### 4.3.1 Patagium geometry and gliding performance

The anatomical and histological data provided here offer new evidence regarding adaptations for gliding in weigeltisaurids. Previous estimates report a mass of 270 g and a wing loading of 102 N m^−2^ for the Eppelton specimen of *Weigeltisaurus* (Evans, 1982) and 107.9 N m^−2^ for *C. elivensis* (McGuire & Dudley, 2011). This led McGuire and Dudley (2011) to suggest that these reptiles might be better considered parachuters rather than true gliders because of their large size, as their wing loading is much larger than in *Draco*. This is in stark contrast with the universal acceptance of gliding capabilities in weigeltisaurids in the paleontological literature (Buffa et al., 2022; Evans, 1982; Evans & Haubold, 1987; Frey et al., 1997; Pritchard et al., 2021; Schaumberg et al., 2007). However, these first estimates were based on the misinterpretation of the patagials as true dorsal ribs (as was commonly accepted at the time) that resulted in an overestimation of the trunk length of these animals.

Here, we estimate a mass of ~135 g for the best‐known weigeltisaurid *C. elivensis* (Table 4) based on the method of Campione and Evans (2012) using the minimum diameter (as a proxy for circumference) of the stylopods of MNHN.F.MAP325a, the only specimen which preserves both a complete humerus and femur. This is half as much as the mass estimate of Evans (1982) for the similar‐sized Eppelton specimen, emphasizing the impact of skeletal reconstruction on mass estimation in these animals. Note that using humeral length as a proxy for individual size, MNHN.F.MAP325a is slightly smaller than the other known *C. elivensis* specimens. *C. elivensis* could thus probably reach a slightly higher mass. Given a planform area (i.e., the projection of the wings and trunk) of ~240 cm^2^ for *C. elivensis* (2022), we estimate a wing loading of about 55.18 N m^−2^ for this taxon (Table 4). This is nearly half of that estimated by McGuire and Dudley (2011), suggesting that *C. elivensis* may have been a better glider than previously thought because gliding performance decreases with wing loading in *Draco* (McGuire, 2003; McGuire & Dudley, 2005, 2011). Our revised mass and wing loading estimates for *C. elivensis* are similar to those of the northern flying squirrel *Glaucomys sabrinus* (*m* = 140 g; WL = 50 N m^−2^; Thorington & Heaney, 1981), which is known to glide at speeds between 6 and 8 m s^−1^ (Scheibe et al., 2006). This supports the idea that *C. elivensis* was an efficient glider (contra McGuire & Dudley, 2011), capable of gliding at similar speeds to that of some extant gliding amniotes. This inferred gliding speed for *C. elivensis* is slightly faster than those recorded for any *Draco* species (McGuire & Dudley, 2005), which conforms to its higher wing loading (Table 4). Additionally, wing loading might have also been kept relatively low in *C. elivensis* (although higher than in *Draco*) due to their very large wingspan with a relatively high aspect ratio compared to *Draco* (Table 4). Aspect ratio is indeed reported to increase with larger individuals and to compensate for increased weight both intra‐ and interspecifically in *Draco* (McGuire, 2003; McGuire & Dudley, 2005).

**TABLE 4:** Aerodynamically significant measurements of the extant flying lizard *Draco volans* and the weigeltisaurid *Coelurosauravus elivensis* (Late Permian, Madagascar). AR equals s^2^ A^−1^; WL equals mg A^−1^, with *g* = 9.81 m s^−2^. “*” indicates estimated measurement of MNHN.F.MAP325a (lectotype) using the method of Campione and Evans (2012) based on stylopod circumference (= minimum diameter * *π*).

Measurement	*Draco volans* ^b^	*Coelurosauravus elivensis*
Snout–vent length SVL (mm)	75.7	~180^a^
Wingspan (*s*) (mm)	70	~350^a^
Planform area (*A*) (cm^2^)	21.50	~240^a^
Aspect ratio (AR)	2.05	5.10
Mass (*m*) (g)	7.25	~135*
Wing loading (WL) (N m^−2^)	29.80	55.18

^a^ Buffa et al. (2022).

^b^ Buffa et al. (2024b).

Lastly, the patagial–gastralial articulation demonstrated here confirms previous interpretations that weigeltisaurids had a low‐wing configuration (Buffa et al., 2022; Pritchard et al., 2021). By comparison to modern aircraft, this configuration would offer more maneuverability but less stability than a high‐wing configuration such as that known for *Draco* (Frey et al., 2003). However, the likely involvement of the forelimb in keeping the patagium unfolded during flight might have also helped in keeping the wing in a dihedral position, which would have stabilized flight and thus at least partially counterbalanced this trade‐off (Frey et al., 2003). Forelimb‐assisted control of the dihedral angle of the wing would have provided a means of stable long‐distance gliding, as well as an option for maneuverability.

#### 4.3.2 Patagial microanatomy and orientation

In extant gliding mammals, the cross section of the long bones that support the patagium is mostly circular, with an extremely thin cortex (Amson & Bibi, 2021). A similar condition has recently been described in the extinct putative gliding reptile *Ozimek*, whose patagium would have also been stretched and supported by its limbs (Konietzko‐Meier et al., 2024). In both cases, this has been interpreted as a way to resist torsion and/or accommodate multidirectional bending loads in extant gliders, although this requires further examination (Amson & Bibi, 2021; Konietzko‐Meier et al., 2024).

**FIGURE 20:**
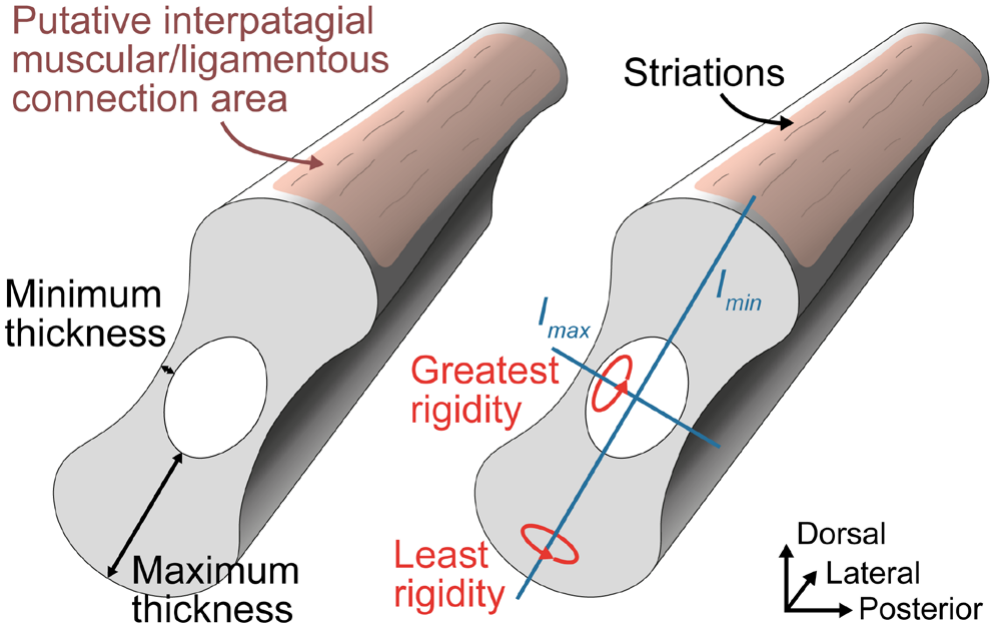
Reconstruction of the middle section of two adjacent patagials showing the cross section of the bone (based on Section 22), its reconstructed structural properties such as the second moment of area *I*, and putative connection areas for interpatagial muscles and/or ligaments. The patagials are slightly posterodorsally angled relative to the vertical plane of the animal, as inferred from the reconstructed structural properties of the bone (see text).

In contrast, the cross section of the weigeltisaurid patagials is ellipsoid throughout the length of these bones and is biconcave throughout nearly their entire length, excluding its most medial and, to a lesser degree, most lateral portions (Figures 14, 15, 16, 17, 18 and 20). This is accompanied by asymmetrical bimodal cortical thickness, especially in the middle part of the shafts, where the cortex can be up to eight times thicker along the long axis of the cross section compared to the short axis (Figure 16). This difference in cross‐sectional shape compared to the long bones of other gliders suggests that, to some extent, the main loads applied onto the patagials during gliding in weigeltisaurids differed from those of other taxa. Indeed, ellipsoid cross sections with bone deposition along a preferred axis are typically found in bones subjected to bending in a given plane (Main et al., 2021). However, this might be confounded by the larger number of supporting elements in weigeltisaurids compared to the limb‐supported patagia of most other gliders, which could permit a different load distribution along the wing. Interestingly, although there is little to no data on the cross section and osteohistology of the ribs of *Draco*, we note that the ribs visible on the wing sections of Russell & Dijkstra (2001: figure 9) show a small degree of eccentricity that is reminiscent of the ellipsoid cross section of the weigeltisaurid patagials. If confirmed, this could further support the idea that the loads applied on a rib‐supported patagium and/or their distribution along the supporting elements are not the same as for a limb‐supported one.

The bimodal cortical thickness distribution of the weigeltisaurid patagials has some implications for their load‐bearing capabilities. A patagial cross section shows an extremely thick cortex along its long axis and a much thinner one along its short axis, especially in the middle portion of the bone (Figure 20). Because of this asymmetry, large differences in the value of the second moment of area *I*, which describes bone distribution along a given axis, are expected. *I*_*max*_, corresponding to the direction of greatest bending resistance (i.e., rigidity), would thus be oriented along the short axis of a patagial cross section, whereas *I*_*min*_, the direction of least bending resistance, would be oriented along the long axis of the cross section (Figure 20). In vertebrate bones, such a shape and difference in *I* is typically found in bones subjected to bending along a preferential direction, along *I*_*max*_ (Main et al., 2021).

In addition, longitudinal furrows run on either side of the weigeltisaurid patagials (excluding the medial and lateral extremities), which give the bone shafts a biconcave cross section, with the concavities distributed along *I*_*max*_ (Figure 20). In engineering, such longitudinal structures serve to increase the rigidity of long hollow tubes (e.g., Al Zand et al., 2020). By analogy, the furrows on the weigeltisaurid patagials would have fulfilled a similar function, further strengthening the resistance to bending of these bones along *I*_*max*_.

As discussed above, weigeltisaurids probably expanded their wing by muscular contraction combined with forelimb‐assisted expansion, as in *Draco* (Dehling, 2017). In this configuration, a chordwise bending load is expected to be applied on the weigeltisaurid patagials. This load is expected to be greatest at mid‐span, where the forelimb pulls on the leading edge, and at the trailing edge, provided that the weigeltisaurid wing is rimmed by a band of connective tissue as is the case in *Draco* (Russell & Dijkstra, 2001). In addition, once airborne, weigeltisaurids likely adopted a posture that sets the leading edge of the wing at a shallow angle of attack relative to the direction of motion, while keeping the wing expanded and slightly cambered for the duration of the flight, as does *Draco* (Khandelwal & Hedrick, 2022). During this phase, the airfoil shape of the wing would be subject to a pressure differential, with the higher pressure applied to its ventral surface pushing the wing upward (Buffa, Salaün, & Cinnella, 2024b; Lau et al., 2023). In *Draco*, it is plausible that the forelimb maintains the integrity of the wing against this pressure differential because the hand extends close to the level of the trailing edge. In contrast, the much larger wingspan of weigeltisaurids precludes such a structurally stabilizing mechanism because the forelimb only extends up to half of the wingspan (Buffa et al., 2022; Pritchard et al., 2021). The lateral half of the wing would thus be subject to a high risk of structural collapse in flight. In light of these reconstructed bending loads, we expect the median half of the patagials to be mainly subjected to bending due to the pressure differential around the airfoil once airborne, with the bending due to the unfolding of the wing counterbalanced by the forelimb. Conversely, the lateral half of the patagials is expected to have been subjected to loads along various directions. As a result, we expect the cross sections of the medial half of the patagials to show a much more marked difference between their directions of greatest and least rigidity, which is confirmed by our thin sections (Figure 16a,b). In contrast, we expect the lateral half of the bones to have more symmetrical cross sections as a trade‐off to resist various loads, which is also confirmed by the data presented here (Figure 16c,d).

In light of all this, we think that, when the patagium is fully extended, the patagials would be oriented in such a way that the short axis of the cross section (*I*_*max*_) is oriented along the chordwise plane, whereas the long axis (*I*_*min*_) is oriented perpendicular to it. Because of wing camber, we expect these planes to be slightly skewed relative to the sagittal and coronal planes of the animal, but this cannot be measured based on the available material (Figure 20). Such an orientation, however, could explain why the patagials are generally preserved with their cross‐sectional long axis exposed (Figures 1, 2, 3, 4, 5, 6, 7, 8), an orientation which, based on the above considerations, would offer only limited rigidity to the patagials against the pressure differential and incoming airflow once airborne. Bringing further support to this, the ribs of *Draco* appear to naturally lie in a similar orientation relative to the wing (LSMUZ herp 81750). However, quantifying this orientation in *Draco* is out of the scope of this paper.

Our reconstruction of the orientation of the patagials inside the patagial membrane suggests an interpretation of the overall curvature of these bones. The Ellrich and Eppelton specimens of *Weigeltisaurus* and MNHN.F.MA324a of *Coelurosauravus* all show a marked lateral bending of the patagials which, once the patagials are re‐oriented, indicates that the patagials showed some degree of dorsoventral curvature (Figures 1, 6 and 8). This would have given the wing an airfoil shape, a prerequisite for lift generation that is found in all extant gliders (Khandelwal et al., 2023; Socha et al., 2015). The aforementioned weigeltisaurid specimens also show some degree of chordwise bending, which is partially obscured by their preservation. Such bending is also seen in the patagial ribs of *Draco*, in which it participates in the folding and unfolding mechanism of the wing (Russell & Dijkstra, 2001).

## 5 CONCLUSIONS

The detailed morphological and osteo‐histological examination of the patagium of the weigeltisaurids *Weigeltisaurus* and *Coelurosauravus* presented here provides new insight into the paleobiology of the world's first gliding tetrapods. The use of computed tomography and laminography, as well as reflectance transformation imaging, provides direct evidence of the one‐to‐one articulation between patagials and gastralia in weigeltisaurids. The lateral end of the gastralia of weigeltisaurids possesses an expanded head with shallow articular cotyles matching the medial head of the adjacent patagial. We also provide evidence that patagials and gastralia are not strictly homologous based on skeletal anatomy and histology, as well as inferred musculoskeletal relationships. Thus, we support the idea that patagials are neomorphs with no known homologue among extant or extinct vertebrates. It is likely, however, that patagials share the same morphogenetic origin as gastralia, originating in the loose mesenchyme adjacent to the body wall.

The data provided here allow for the first anatomy‐ and histology‐based reconstruction of the musculoskeletal organization of the wing in weigeltisaurids. It is likely that the neomorphic patagials were embedded in the M. obliquus externus group and that this muscle was modified for the operation of the patagial skeleton, structurally similar to the condition in the extant flying lizard *Draco*. These muscles may have contributed to the unfolding of the patagium, which was likely supplemented by hooking the manual claws onto the scales of the leading edge of the patagium, as does *Draco*. This would have provided sufficient means to keep the patagium unfolded and under tension while gliding, as well as a way of keeping the wing at a dihedral angle, thereby offering a means to control stability and maneuverability while airborne. Wing folding may have been enabled by muscular and tendinous connections between the individual patagials, generating intrinsic elastic tension toward a folded state, as in *Draco*. Furthermore, the asymmetrical bimodal cortical thickness distribution seen in the cross sections of the weigeltisaurid patagials suggests that these bones were sufficiently stiff across the wingspan to prevent patagial collapse once airborne. The new data presented here are thus critical for our understanding of the wing structure and gliding mechanism in weigeltisaurids, paving the way for future morphofunctional or biomechanical studies on the locomotion of the world's first flying tetrapods.

## AUTHOR CONTRIBUTIONS

V.B. designed the study, examined the material, collected and interpreted the data, and wrote the first draft. T.K. and M.Z. acquired the Computed Laminography data. J.G. and M.G. edited the BoneProfileR code that underlies this study. J.G., M.G., J.S.S., M.L., and D.F. interpreted the data. All authors contributed to the discussion and approved the final manuscript.

## Data Availability

The data underlying this article, including the reflectance transformation imaging interactive pictures, and the X‐ray tomographic and laminographic data, are available through the RADAR4KIT repository at https://doi.org/10.35097/g5n11t5gencnav5s.
